# Learning-Induced Changes in Attentional Allocation during Categorization: A Sizable Catalog of Attention Change as Measured by Eye Movements

**DOI:** 10.1371/journal.pone.0083302

**Published:** 2014-01-31

**Authors:** Caitlyn M. McColeman, Jordan I. Barnes, Lihan Chen, Kimberly M. Meier, R. Calen Walshe, Mark R. Blair

**Affiliations:** 1 Department of Psychology, Simon Fraser University, Burnaby, Canada; 2 Cognitive Science Program, Simon Fraser University, Burnaby, Canada; 3 Department of Psychology, University of British Columbia, Vancouver, Canada; 4 Department of Psychology, University of Edinburgh, Midlothian, United Kingdom; University of Plymouth, United Kingdom

## Abstract

Learning how to allocate attention properly is essential for success at many categorization tasks. Advances in our understanding of learned attention are stymied by a chicken-and-egg problem: there are no theoretical accounts of learned attention that predict patterns of eye movements, making data collection difficult to justify, and there are not enough datasets to support the development of a rich theory of learned attention. The present work addresses this by reporting five measures relating to the overt allocation of attention across 10 category learning experiments: accuracy, probability of fixating irrelevant information, number of fixations to category features, the amount of change in the allocation of attention (using a new measure called Time Proportion Shift - TIPS), and a measure of the relationship between attention change and erroneous responses. Using these measures, the data suggest that eye-movements are not substantially connected to error in most cases and that aggregate trial-by-trial attention change is generally stable across a number of changing task variables. The data presented here provide a target for computational models that aim to account for changes in overt attentional behaviors across learning.

## Introduction

The visual modality is a primary source of information from which our understanding of the world arises. We learn to navigate through this world by prioritizing relevant sources of information to which we further allocate mental resources. The relevance of certain properties of the visual scene changes based on the goals of the observer; meaning that our visual-cognitive system must be able to respond to changes in both the task at hand and the scene itself. This question of how the human cognitive architecture is able to flexibly respond to a complex, dynamically changing visual environment is a defining problem for the psychological sciences [1]–[2]. Cognitive flexibility depends to a large degree on the capacity to make quick and efficient classifications of objects in the environment. Rapid classifications, in turn often depend on learning to attend to the features of objects that most effectively differentiate alternatives. For instance, when driving towards an intersection, deciding whether to stop the car critically depends on learning to attend toward the information conveyed by traffic lights. Because of the complexities of real world decision-making, we extract the basic elements of these decisions to study them in controlled tasks in the laboratory. In this paper we use eye-tracking to explore how allocation of overt attention changes over the course of learning by providing data from ten such classification experiments. The resulting data are made available to inform the advancement of theories of goal-directed attention and category learning.

Eye movements are an effective index of visual selective attention due, in part, to the close coupling of attentional and oculomotor processing at a neurological level [3]. The strategy of tracking attention using eye movements has been leveraged in a number of studies, which have found that oculomotor activity is sensitive to small task variations [4] and prior knowledge [5]–[6]. In studies of sport expertise it is typically found that skilled players allocate their gaze to locations at which the ball is likely to land [7], in addition to allocating gaze in ways that minimize the effects of inattentional blindness [8]. The coupling of the allocation of gaze and online performance [9] was also shown in several landmark studies of gaze-allocation during tasks such as the preparation of tea and sandwich making [10] and a change in gaze paths following variable instruction sets [11]. These studies show that eye movements capture the goal-directed attentional processing of participants in a variety of tasks and that effective attentional deployment is, at least in part, a learned skill [12]–[13].

This idea of *learned attention* has been explored in several computational models of category learning [14]–[16], a field that has, perhaps, the most formalized relationships between attention and learning. In the category learning literature, attention is typically defined as the preferential weight assigned to the important dimensions of a category represented in formal models. These formal models do not predict eye movements or other observable indices of attention, rather, attention is an abstract concept that increases the likelihood to make a particular category decision based on the input to the model. Error-driven algorithms, such as back-propagation, are the most commonly implemented attentional learning mechanisms in formal models. In these algorithms, the degree of change in the model's attention weights is proportional to the degree of mismatch between model output and the correct response: larger weight changes result from a larger discrepancy between model output and target values. This behaviour is a basic consequence of the classic delta-rule formulation, *Δw_i,j_ = α(t_j_-y_j_)x_i_*, where *w_i,j_* is the weight connecting hidden (or input) unit *x_i_* with the output unit *y_j_*, in a supervised network model where *t_j_* is the teacher and *α* is the learning rate. This is the standard basis for error-driven learning. One problem with using the delta rule to formalize notions of human attention changes in categorization tasks is that the error generated by the model cannot be measured directly in human participants, as there is no known method to measure the values of internal dimension weights. As such, a hypothesis like ‘attention change is tied to error’ is hard to test, since error is a discrete measure in human data and error in model attention weights is a continuous value. Further, error in formal models is disembodied and abstract in that the continuously valued prediction cannot be reduced to human performance errors. This problem extends more generally, in that attention weights are not observable in human learners over the course of the learning curve, in response preparation, or other important parts of categorization that models of category learning aim to fit. Despite pervading formal models, attention weights are an ethereal, immeasurable stand-in for attentional processes preceding category choices. In contrast, eye movements provide an observable measure of attention which can be leveraged to understand the deployment of attention to stimulus features preceding category choices [17]–[18].

An additional part of the problem of comparing human performance to that of models is the paucity of data from which to base theories. To make an effort to extend the data available, we re-analyze a number of published experiments (using different measures), and also report some new data that has not yet been seen. Given the variation seen in eye-movements based on very subtle tasks or environment changes, constructing a model to fit one or two experimental manipulations does little to advance a greater understanding of selective attention during learning more generally (see [19] for additional discussion on this topic). In the spirit of developing a point of comparison between humans and popular computational models of learning and attention, recent work was undertaken to compare the two using an early measure of attention change after error and correct trials in a category learning task [20]. The results show a large disparity between formalizations of attentional learning and actual human behavior. The difference between observable measures of attention and the formal predictions point to a need to investigate the relationship more comprehensively.

Although there is evidence to suggest that improved attention patterns are related to the change in accuracy, there are further concerns, beyond the simulation data, of relying on error reduction algorithms that target attention weights to understand attention change. Error-driven theories have been shown to be problematic, as overt attention changes are observed well after participants stop making errors during learning [17], [21]. This suggests a disconnect, or a mediating factor that separates attention from response patterns, in contrast with the purportedly tight coupling between the attention and accuracy expected in formal models relying on error reduction algorithms. It could be, as Rehder and Hoffman suggest, that there are simple factors like strategic delays or spatial translation times that explain these disconnects, but regardless of the source of the dissociation, it is important to acknowledge that attention patterns predicted in formal theories are often not commensurate with human data. We propose that collecting the multifaceted body of evidence of attention patterns in human learners described here is a necessary precursor to developing a model that is able to realistically account for attentional learning. The data presented here come from experiments that use similar category structures as those that have been used to test formal models [14] with the goal being to provide a novel look at the underlying assumptions, and a more plausible behavioral basis for models of category learning.

The following ten experiments are examined using five measures: accuracy, probability of fixating irrelevant information, the number of fixations to category features, and two novel measures that describe the deployment of attention in a category learning task, which we call Time Proportion Shift (TIPS) and Error Bias. Separate measures using these data have been reported elsewhere, but the analyses reported here have not been reported using these experiments. We report classification accuracy, a standard index of knowledge acquisition, to convey the extent of participants' knowledge of the category structures with which they are presented. The probability of fixating irrelevant information reflects one dimension of the attentional expertise of the participants. This measure captures moments of uncertainty, in that any eye movement to fixate an irrelevant feature is one that the participant could have used to examine task-relevant areas of the environment. The number of fixations to category features elaborates on attentional efficiency in that a larger number of fixations than there are relevant dimensions in the stimuli is redundant and reflects some level of inefficiency. As we show in this report, there are cases where participants are unlikely to fixate irrelevant information, but still deploy a relatively large number of fixations to the stimulus features, reflecting an inefficient use of eye movements.

TIPS reflects the aggregate changes in eye-movements, trial-to-trial. It is quantified by the sum of the differences in the proportion of fixation time to the features of a stimulus in a categorization task. Formally stated, we let φ*_i,t_* be the total amount of time spent on feature *i* during trial *t*. We then calculate the proportion of time spent on feature *i* during trial *t*, 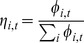. The TIPS from trial *t*-1 to trial *t* is defined simply as the total absolute difference between η_i,t_ and η_i,t-1_ for each *i*, namely:
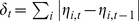
(1)


The range of the TIPS score for any pair of trials is 0 to 2. We can see this most simply in the case of a two featured stimulus, where the maximum change in attention would be all gaze allocated to a particular feature on trial *t*-1 followed by all gaze allocated to the alternative feature on trial *t* (e.g. |(0–1)|+|(1–0)| = 2. In our experiments, features are considered for their relevance to the category response rather than their spatial location. This measure captures the stability of between-trial attentional allocation, which might act as an effective aggregate complement to recently developed measures that explore the stability or strengths of within-trial attentional patterns [22]. From earlier work exploring eye movements in category learning tasks we expect that TIPS will decrease over time as participants start to make more automated movements. For instance, it was found that attentional optimization improves over learning, as measured by decreases by both numbers of fixations and fixation durations to irrelevant information [17], [21]. Additionally, recent work [22], has suggested that participants in categorization tasks develop strong consistent eye movement patterns over the course of learning, as measured by tendencies to look at information in the same order, even if that order is sub-optimal in terms probability or information gain. As eye movements become more efficient, trial-by-trial attention changes should decrease while the participant is developing a consistent, efficient strategy, resulting in a lower TIPS score. Fixations durations to features may show a level of systematic variability that makes TIPS differ in its patterning than what might be easily predicted from the trends in other efficiency measures, and so pairing TIPS with other efficiency measures builds a multi-pronged profile for attentional data.

From the TIPS measure we develop a second measure of attention change, this one in response to predictions that tie attention change and accuracy together. We do this, not only to allow comparison with computational models of learning and attention, but also as a way of demonstrating the capacity of this kind of data to yield task sensitive descriptions in terms of aggregate eye-movement data. To calculate Error Bias, we let *e* denote a trial that follows an error trial, and *c* denote a trial that follows a correct trial. The mean TIPS after error and the mean TIPS following correct trials over the course of the experiment are, 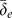 and, 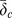 respectively. The raw Error Bias score for each participant is then defined as,
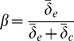
(2)which is then normalized to produce Error Bias β′ = 2(β−1/2) so that the score ranges from −1 to 1. An Error Bias of −1 indicates that there was no attention change following error trials since TIPS = 0 after error; rather the entirety of the attention change in the experiment follows correct trials. Conversely, an Error Bias of 1 means that all attention change followed error trials. These two novel measures capture different elements of attentional learning in categorization tasks.

We show a more complex relationship between learning and attention than has previously been acknowledged in the categorization literature, by presenting data from ten experiments. Some of the findings reported here are new analyses on existing data. All such cases are clearly marked during the description of the experiment. A number of manipulations influence the attention measures in ways that have not yet been reported: the complexity of a task, the types of stimuli, the length of the experiment, and the medium of access all work to influence how participants attend information. In the interest of presenting a robust dataset that can be replicated and examined in laboratories without access to eye gaze data, and to demonstrate convergent results across modalities, we describe an experiment where mouse movements are used as indices of attention (see Experiment 10). In this regard, laboratories that are not equipped with eye trackers are also able to tap into the fine-grained, temporal nuances of attentional deployment, and how it changes over the course of learning; we also discuss some of the differences between hand-movements and eye-movements that researchers should bear in mind when comparing these measures. Unearthing all of the complex relationships between all of these measures, however, will be an ongoing effort and the large dataset provided here serves as an initial step in that pursuit.

## Experiments

This section describes the ten category learning experiments, all of which measure the overt allocation of attention to stimulus dimensions by tracking eye movements (or mouse drags, Experiment 10). These experiments are presented in a manner that increments the task complexity and includes important category structures found in the categorization literature. In each case we report five measures of learning and attentional performance: accuracy, the probability of fixating irrelevant information, the number of fixations to category features, the Time Proportion Shift (TIPS) and the degree of Error Bias. Some of the present work, as indicated, is a reanalysis of eye-movement data from earlier experiments that have been described elsewhere for other research goals based on different measures.

### Ethics Statement

These data were collected with approval from the Office of Research Ethics at Simon Fraser University, project number 37046. All participants provided written informed consent at the beginning of the experiment, and were provided with a written debrief form after the experiment.

### Experiment 1. Categories Defined Using Simple Conjunction

**Figure 1:**
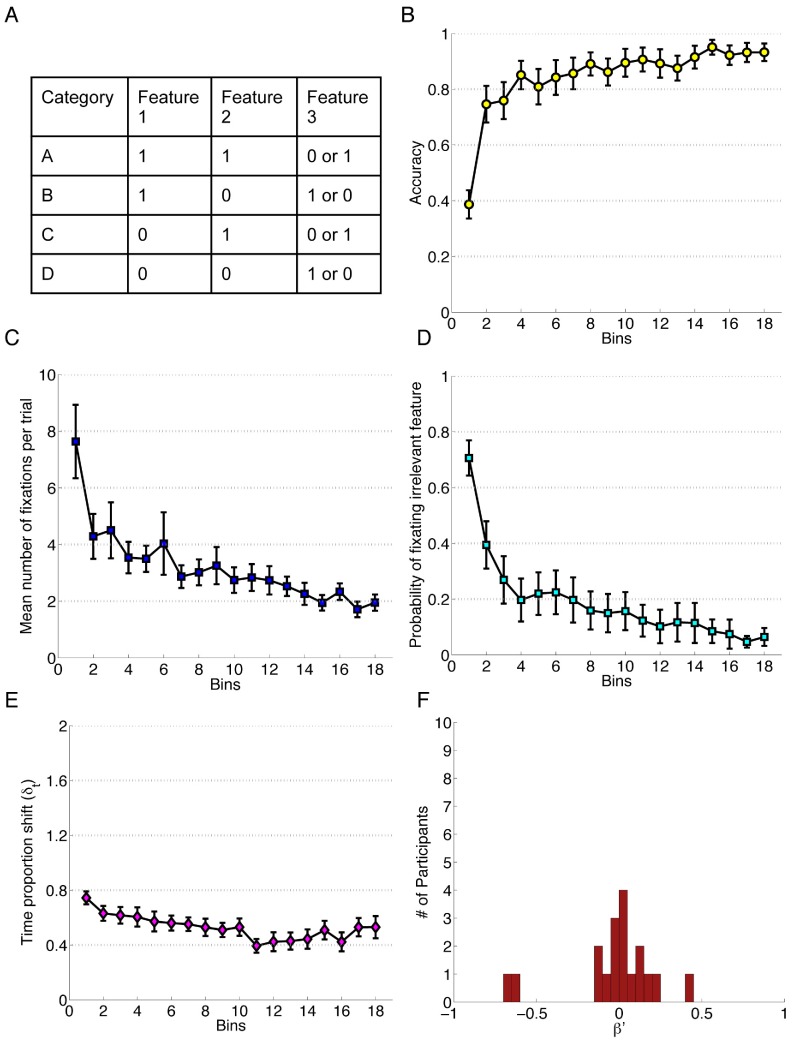
Data for Experiment 1. Each bin reflects the average of 20 trials and error bars represent standard error of the mean. A) The category structure. B) Performance accuracy. C) Mean number of fixations per trial. D) Probability of fixating the irrelevant feature. E) Time proportion shift, δ_t_. F) Error Bias, β′.

Experiment 1 was a simple categorization task, in which participants used a two-dimensional conjunction rule informed by two of the three stimulus features (Figure 1A). The two features that were used in the category decision were the *relevant* features, while the third was an *irrelevant* feature. These data were taken from one of the experimental conditions of McColeman, Ancell and Blair [23], and the raw data were made available through SFU Summit under “Category Learning with Imperfect Feedback”. Unlike subsequent experiments reported here, participants were told that only two features would be relevant to the category choice. However, the participants have to learn which of the three features, or which of the feature locations were relevant.

#### Methods

Twenty-two undergraduates at Simon Fraser University received course credit for their participation. The data from two participants were excluded from analysis for failure to meet the eye gaze quality criteria of at least 70% of the trials exceeding 75% gaze collected. One participant failed to reach the learning criterion of 9 consecutive correct trials and was also excluded from analysis. Data were analyzed for 19 participants.

#### Stimuli and category structure

Participants learned a four-category task using stimuli with three binary-valued features. The configuration of two of these features determines the category of the stimulus, while the third feature is irrelevant (Figure 1, “Data for Experiment 1”, Panel A). Stimuli were fictitious microorganisms in which the three features, appearing as organelles spanning 1.3° of visual angle, were located equidistant from each other and from the center of the image (shown in Figure S1, “Stimuli for Experiment 1”) separated by 10.6°. As is the case for all subsequent experiments, the assignment of location and the relevance of the organelles were counterbalanced between subjects, but the location and relevance of the organelles to making a category choice remained constant for each participant. Unless noted otherwise, in all eye-tracking experiments, eye gaze was recorded using a Tobii X120 eye-tracker sampling at 120 Hz with a spatial resolution of 0.5° and fixations were defined by transforming raw gaze data using a modified dispersion threshold algorithm with a spatial dispersion threshold of 1.9° and a duration threshold of 75 ms [24]. These experiments also used a nine-point calibration technique to establish the position of participants’ eyes at the beginning of the task.

#### Procedure

At the beginning of the experiment, participants were introduced to a fictitious space laboratory, and told that they were to view a series of organisms that belonged to four species. Each species produced its own mineral, which is the basis for participants’ category responses. The experiment consisted of 360 trials in blocks of 24 trials, where each block presents a random ordering of three of each of the eight possible stimuli. Responses were madeta using the four trigger buttons on a Logitech game pad. Following a response, the participant’s selection was displayed alongside a feedback screen displaying the correct answer (‘sodium’, ‘lithium’, ‘potassium’ or ‘calcium’) and the re-presentation of the stimulus from that trial. In this experiment, fixations were considered as fixations to features if they fell within 2.98° of a feature’s center.

#### Results

For each of the experiments in this report, we provide a mixed effects logistic regression (LMER) for measures that yielded binary responses trial-to-trial (accuracy and the probability of fixating irrelevant features); and a within-subjects analysis of variance (ANOVA) on the number of fixations and the TIPS scores. Each analysis contained four levels of the within-subjects factor, Block. Each Block (1–4) captured the average score for the corresponding quarter of the experiment. In employing this technique we were able to compare across a number of experiments of varying trial lengths. To provide a clear picture of the underlying data that informed these analyses, data were visualized in bins of 20 trials each for easy comparison between experiments, shown in Figure 1. Thus, for all of the experiments reported here, one bin on a plot reflects the average of 20 trials; and one level of the within-subjects factor Block in the ANOVA reflects 25% of the total number of trials in the experiment. Text S1, “Four Block Experiment Data”, describes the conditional means for the data used in the statistical analyses. These measures were calculated based on data from trials where the eye-tracker successfully recorded over 75% of gaze positions, a standard used consistently throughout all of the experiments. Post-hoc analyses were performed using Tukey’s HSD (α = .05) with corrections for sphericity [26] where necessary (Text S1, “Four Block Experiment Data”).

In this experiment, each Block (1–4) of each one-way within-subjects analysis contained 90 trials, or 4.5 bins of each plot (Figure 1, A–E). The first analysis was conducted on participants’ response accuracy with Block as a fixed effect and Subjects as a random effect. To perform this analysis, we used the lme4 package in the freely available statistical software, *R* [25]. We find that Block is a significant predictor for accuracy, β_block_ = 0.78, estimated *z* = 16.32, *p<*0.001, indicating that the model constructed with Block is a stronger predictor of accuracy than a model comprised of only the intercept. From this model, we can infer that accuracy increased with block. Full model specification is available in the supplementary material (along with the conditional means and standard deviations, Text S1, “Four Block Experiment Data”).

Concurrent with learning the categories, participants learned to use more efficient attentional patterns, as reflected in two measures: probability of fixating the irrelevant feature, such that more efficient patterns direct attention toward the irrelevant information less often [21] and the average number of fixations they made during a trial, which is related to repeat fixations to features already viewed. A linear effects model to predict participants’ probability of fixating the irrelevant feature revealing Block to be a significant predictor, β_block_ = −1.23, *z* = 19.56, *p*<0.001. The model indicates that the probability of fixating irrelevant information decreases over time. An ANOVA conducted on participants’ average number of fixations per trial revealed a significant main effect of Block, *F*(1.67, 30.04) = 13.16, *p*<.001 after Greenhouse-Geisser correction, *η_G_^2^* = .19 [27]. Post-hoc analysis revealed that Block 1 had significantly more fixations than all other Blocks. Additionally, significantly more fixations were made in Block 2 compared to Block 4 (see Figure 1, Panel C).

An ANOVA was conducted on participants’ TIme Proportion Shift (TIPS) scores (δ_t_). Recall that this number reflects the degree to which participants alter the proportion of time spent fixating each of the three features from one trial to the next, and acts as an index of participants’ propensity to change their information access patterns. That is, a lower TIPS score indicates a more stable fixation pattern. One participant was excluded from the analysis of TIPS data due to one cell of missing data: unlike our other measures, this relies on two successive trials of clean data, so problems with either trial such as insufficient gaze collected by the eye-tracker invalidates that particular data point. We found a significant main effect of Block on TIPS, *F*(1.74, 29.52) = 5.83, *p = *0.007 after Greenhouse-Geisser correction, *η_G_^2^* = 0.14. Post-hoc analysis suggests participants were allocating attention more constantly as learning improves, with a significant difference found only between Blocks 1 and 3 (see Figure 1, Panel E; and Text S1, “Four Block Experiment Data”).

Finally, we investigated the extent to which participants tended to engage in attention shifts in response to correct or incorrect trials by examining their Error Bias (*β*′) scores. Recall that this score is an aggregate of behavior over the entire experiment, and that a score of −1 indicates that none of the attention shift in the experiment follows error and a score of +1 indicates that all of the attentional shifting occurs after an error trial. A single sample t-test detected no significant difference from 0 in the Error Bias distribution (*M* = −0.02, *SD* = 0.27), *t*(17) =  −0.31, *p = *0.759 (Figure 1, Panel F). We thus find no evidence to suggest that participants are more likely to shift their attention following a correct trial or an error trial.

### Experiment 2. A Second Case of Simple Conjunction, with a Speed/accuracy Manipulation

Building on the data derived from simple conjunction rule use, this experiment had a similar design to that of Experiment 1, however the instructions were worded such that each of the two conditions prioritized either speed or accuracy in participants’ responses. It was expected that the number of fixations and the information access strategy would differ between groups depending on the priority to respond either quickly or accurately. The data from this experiment and Experiment 7 are publicly available through the SFU Summit at summit.sfu.ca/collection/94 under “Speed Accuracy Trade-Offs in Category Learning”.

#### Participants

Sixty-nine undergraduates participated in this experiment. Four were excluded from analysis due to bad gaze quality, and an additional fifteen were excluded for failure to meet a learning criterion of twelve consecutive correct trials. Data were analyzed for 50 participants.

#### Stimuli and category structure

The category structure for this experiment was the same as in Experiment 1: the values of two of the features were the basis of four categories, while a third feature was irrelevant (Figure 1, Panel A). These stimuli were mock animal cells, shown in Figure S2, “Stimulus and feature images for Experiments 2 and 7”.

#### Procedure

At the beginning of the experiment, participants were assigned to either a speed-emphasis condition (25 participants) or an accuracy-emphasis condition (25 participants) wherein either the speed or the accuracy of responses were prioritized for the participant by the instructions that introduced the experimental task. A break at the end of each 20 trials indicated to a participant their average response speed and accuracy from those past 20 trials, a reminder to perform quickly or accurately (corresponding to the condition assignment), and the number of blocks remaining in the experiment. The trial procedure was the same as Experiment 1 where participants were presented with a stimulus, made their self-timed response, and then received feedback along with a re-presentation of the same stimulus. Feedback in this experiment differed slightly from the earlier experiment in that a square in each corner of the screen corresponded to the physical location of the response buttons on the gamepad. The correct square was highlighted in green and, if the participant was wrong, the incorrect square was highlighted in red to provide information about response accuracy. The experiment terminated after 300 trials. Technical specifications were the same as in Experiment 1. A feature was considered fixated if the fixation fell within 3.20° visual angle of the center of the feature.

#### Results

**Figure 2:**
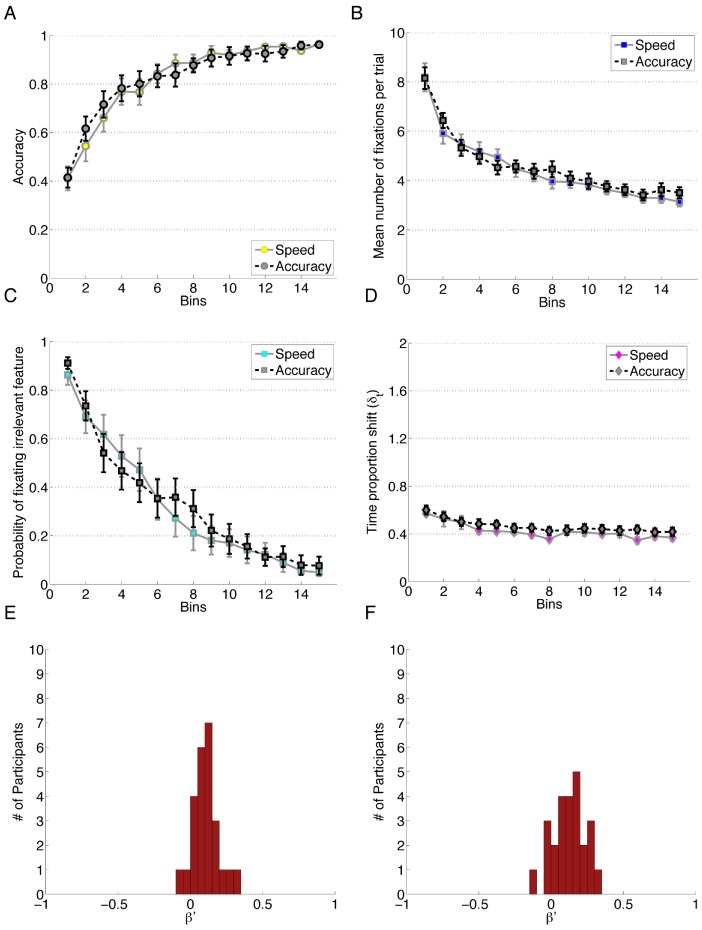
Data for Experiment 2. A) Performance accuracy. B) Mean number of fixations per trial. C) Probability of fixating the irrelevant feature. D) Time proportion shift, δ_t_. E) Error Bias, β′, for speed instruction condition. F) Error Bias for accuracy instruction condition. Each bin reflects the average of 20 trials.

As in Experiment 1, data are presented in Blocks, where each level of the Block factor represents 75 trials or 3.75 bins of Figure 2; additionally, we added Instruction Condition (Speed, Accuracy) as a between-subjects factor in the ANOVA analyses, and as a fixed effect in the LMER models constructed from the accuracy and probability of fixating irrelevant features. The means and standard deviations are available in Text S1, “Four Block Experiment Data”. The first model shows that Block is a significant predictor of accuracy in this experiment, β_block = _1.06, *z* = 9.48, *p*<0.001, but that Condition, *z* = 0.34 *p* = 0.76, and the interaction between Condition and Block, *z* = 0.05, *p* = 0.75 are not. Although there was a decrease in the number of errors over time, there was no detectable influence of the Instruction Condition, indicating that the experimental manipulation prioritizing accuracy versus speed was unsuccessful. Further exploration is necessary to make any direct claims about why this might be the case, but it is likely that improving accuracy is a necessary first step to improving efficiency in a categorization task and participants were self-imposing an accuracy priority regardless of the experiment instructions. Additionally, the failure to elicit a difference between groups is partially attributed to the exclusion criteria: participants who performed effectively enough to be identified as learners were the only ones included in the analysis. Since prior research has consistently shown that there is a trade off between speed and accuracy, it is likely the instruction manipulation was not strong enough to overpower the priority that these participants placed on accurate performance. As the differences between the two groups were non-significant, we collapsed the data across the two conditions to report the change in all fifty subjects over the course of the experiment. The resulting conditional means and standard deviations are available in Text S1, “Four Block Experiment Data”. Though we failed to elicit an effect of the speed-accuracy trade-off, these data provide additional evidence for the behavior of the measures reported throughout this paper and are provided to extend the public dataset of eye movements and learning behavior.

Using a separate linear mixed effects model, we saw that the probability of fixating irrelevant was predicted by Block, β_block_ = −2.35, *z* = −10.48, *p*<0.001 but not by Condition (*z* = −0.74, *p* = 0.81) or the interaction between Block and Condition (*z* = 0.74, *p* = 0.46). An ANOVA conducted on the number of fixations showed significant changes over the duration of the experiment. The ANOVA revealed a large effect of Block, *F*(2.04, 98.08) = 50.83, *p*<.001 after Greenhouse-Geisser correction, *η_G_^2^* = .30; but no main effect of Instruction Condition, *F*(1, 48) = 0.24, *p = *0.63 and no interaction between the two factors, *F(*2.04, 98.08) = 0.21, *p = *0.82. As with above, the between subjects conditions were collapsed to examine the overall trend over the course of the experiment, and so the post-hoc analysis on the number of fixations to features was conducted using the data from fifty participants. These analysis showed that all four of the Blocks were different from one another. That is, the number of fixations decreases over the course of the experiment and did not level out by the last quarter of the trials.

A final ANOVA with the same factors was conducted on the Time Proportion Shift (TIPS) scores. Excluding one participant for missing data, there was a small but significant effect of Block on attention change, *F*(2.98, 140.18) = 7.29, *p<*0.001, after Huynh-Feldt correction, *η_G_^2^* = .05. The speed versus accuracy manipulation failed to elicit a difference in TIPS, as there was no main effect of Instruction Condition, *F*(1, 47) = 2.40, *p = *0.13. The two factors did not interact, *F(*2.98, 140.18) = 0.42, *p = *0.74, indicating the absence of a secondary influence of the instruction manipulation over the four blocks. Post-hoc analysis on the collapsed data reflect differences between Block 1 and all of Blocks 2–4, as well as a difference between Blocks 2 and 4. These findings suggest that TIPS decreases through the first half of the experiment, at which point the attention change remained constant. The degree of Error Bias in these conditions was not significantly different, *t*(48) = 0.63, *p = *0.534, suggesting that both the Speed and Accuracy conditions were similarly non-zero in their Error Bias distributions. Consequently, the Error Bias data points were combined to do one single sample t-test against the null hypothesis of 0 Error Bias, which showed an Error Bias (*M = *0.10, *SD* = 0.10) significantly greater than zero, *t*(49) = 7.69, *p<0.*001, *d* = 1.09, reflecting an overall bias to change attention patterns following an error trial.

Visual inspection of Figures 1 and 2 indicate that of the six measures, two exhibit less optimal outcomes in Experiment 2 relative to Experiment 1. Although the experiments were based on the same simple category structure, a potential source of the difference is the stimuli used in this experiment. Although it is beyond the scope of this paper, it is important to note that the properties of the visual environment will influence the visual attention patterns of a participant. The discrimination of the feature values was more challenging in this experiment than in Experiment 1, which is thought to be a source of minor variation between the two experiments (S1, S2; “Stimuli for Experiments”). A second influence on performance is the information provided to participants in the instruction set, in that Experiment 1 identified that there were only two relevant dimensions while that fact is left to the participants to learn on their own in Experiment 2.

### Experiment 3. Simple Conjunction Rule Applied to Continuous Dimensions

This is the third experiment in which we explore the two-feature conjunction rule. In contrast to the previous experiments, this experiment used continuous, rather than binary-valued, features. As in the previous two experiments, two features were relevant for classification and one feature was never diagnostic of the correct category. The rule-based structure of this category can be thought of as a continuous-feature version of Experiments 1 and 2. Details pertaining to the participants in the study, and the specifications of the stimuli used are available in Chen and colleagues’ paper (Experiment 2) [22]. The analyses below were not described in their report, and the exclusion criterion differs slightly in that for these analyses participants who achieved a learning criterion of nine correct trials in a row were included for analysis, leaving a total of 24. Unlike the previous experiments, a Tobii X50 eye-tracker sampled eye movements at 50 Hz. For Experiments 3, 4, 5 and 6, fixations occurring within 3.73° visual angle of the center of a feature were recorded as fixations to that feature. The raw data for Experiments 3 and 4 can be accessed at summit.sfu.ca/collection/94 under the title “Four Category Continuous Dimension Learning”.

#### Results

**Figure 3:**
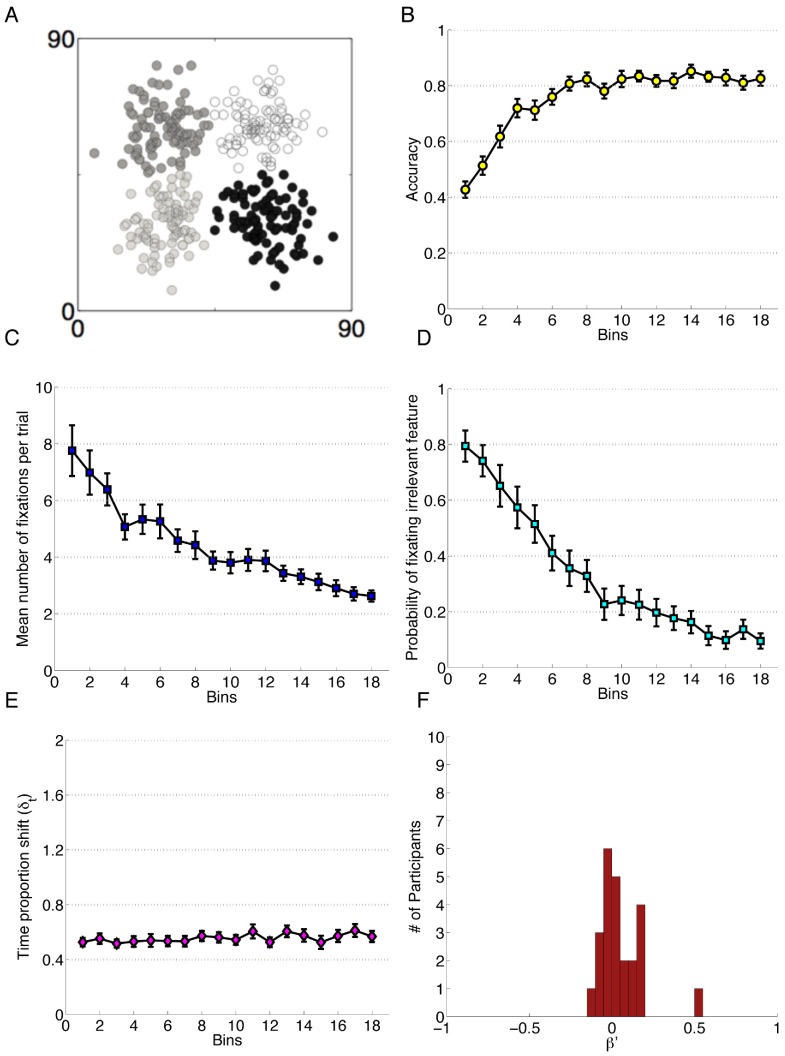
Data for Experiment 3. Each bin reflects the average of 20 trials and error bars represent standard error of the mean. A) The category structure. B) Performance accuracy. C) Mean number of fixations per trial. D) Probability of fixating the irrelevant feature. E) Time proportion shift, δ_t_. F) Error Bias, β′.

In this experiment, each level of within-factor Block represents the aggregate accuracy over 90 trials (4.5 bins of Figure 3). The first analysis was conducted using linear mixed effects modeling (LMER), which revealed Block to be a significant predictor of accuracy, β_block_ = 0.54, *z* = 20.37, *p*<0.001. We also analyzed the two measures of attentional efficiency to see if attention patterns would become more efficient over time like in Experiments 1 and 2. A linear mixed effects model constructed using the probability of fixating irrelevant information indicated that Block was a significant predictor, β_block_ = −1.44, *z* = −36.02, *p*<0.001, demonstrating increased efficiency in attentional allocation across the duration of the experiment. We also found a large, significant main effect of Block on the number of fixations participants made per trial, *F*(1.84, 42.26) = 53.33, *p*<.001 after Greenhouse-Geisser correction, *η_G_^2^* = 0.33. Post-hoc analyses indicated significant differences between all Blocks, suggesting participants continued to decrease their number of fixations over the entire experiment (Figure 3B).

The final ANOVA conducted on this experiment data did not show an effect of Block on TIPS, *F*(3, 69) = 1.38, *p = *0.26, reflecting no difference in attention change over the course of the experiment. A single sample t-test, *t*(23) = 1.76, *p* = 0.092, revealed no significant Error Bias (*M* = 0.05, *SD* = 0.14), indicating attention change after error trials was not detectably different from change after correct trials. The means and standard deviations for all of the reported data are available in Text S1, “Four Block Experiment Data”.

### Experiment 4. An Information Integration Category Learning Experiment

The experiments examined so far used a rather simple rule-based structure. We see a general consistency across the five measures in rule-based tasks exhibited in the above analysis. However, the task structure used in Experiment 4 made creating simple rules and decision thresholds more difficult for the participant. We included this experiment in our analyses to expand on our findings of the five reported measures beyond a simple rule conjunction task as well as to relate these findings to other important contributions in the categorization literature. This four-category structure was very similar to the four-category information integration category structure used by Maddox, Filoteo, Hejl, and Ing [28] but here, features are separated in space. Other data from these participants and the precise distributional parameters were described in Chen and colleagues’ Experiment 2 [22]. As in the previous experiments, two features were relevant for classification and one feature was never diagnostic of the correct category. The procedure, learning criterion and gaze collection methods were identical to Experiment 3 above.

#### Results

**Figure 4:**
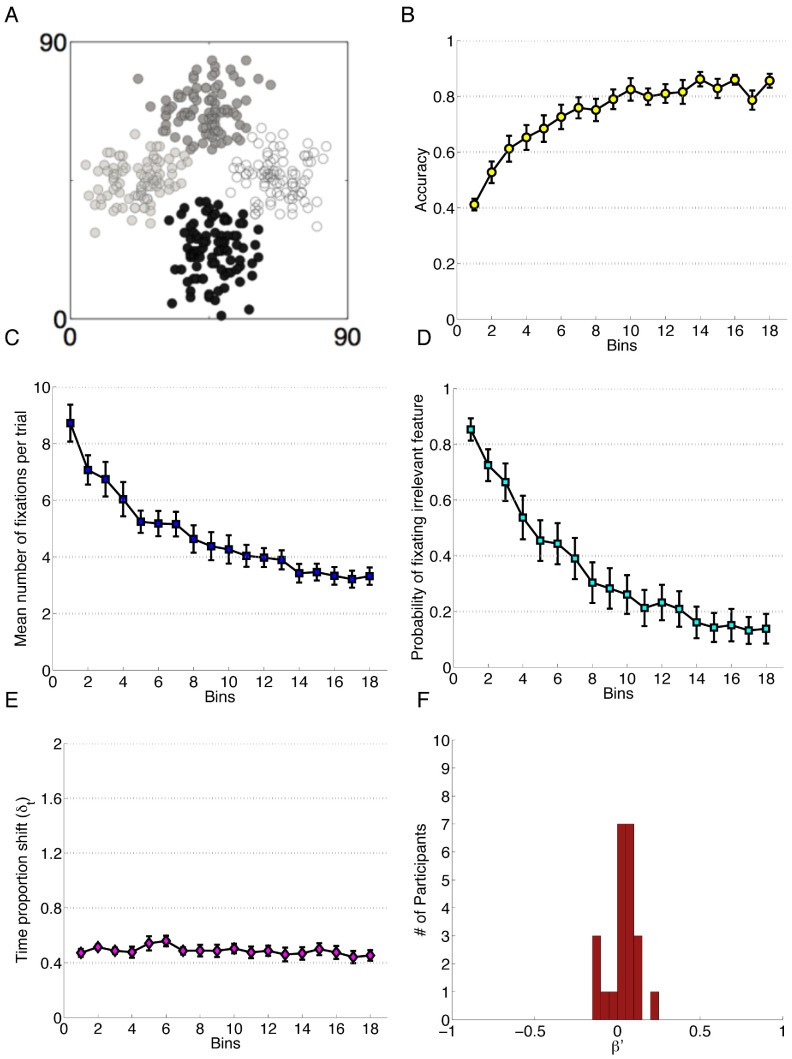
Data for Experiment 4. Each bin reflects the average of 20 trials and error bars represent standard error of the mean. A) The category structure. B) Performance accuracy. C) Mean number of fixations per trial. D) Probability of fixating the irrelevant feature. E) Time proportion shift, δ_t_. F) Error Bias, β′.

As before, we conducted a series of analyses on the data of interest using within-subjects factor Block (1–4). Here, each Block reflects performance over 90 trials or 4.5 bins of Figure 4. The means and standard deviations for the data are available in Table 4 in Text S1, “Four Block Experiment Data”. A linear mixed effects model was constructed using the observed accuracy data, and showed that Block was a significant predictor of performance, β_block_ = 0.44, *z* = 17.05, *p*<0.001 reflecting an improvement in performance over time (Figure 4B).

Again, participants exhibited more efficient attentional patterns over the duration of the experiment. In evaluating the effect of Block on the probability of fixating the irrelevant feature (Figure 4D) we found that Block was a significant predictor in a linear mixed effect model, β_block_ = −1.22, z = −35.72, *p*<0.001, reflecting more efficient distributions of attention as the experiment proceeded. An ANOVA revealed a large significant main effect of Block on average number of fixations per trial (Figure 4C), *F*(1.69, 37.13) = 34.11, *p*<0.001 after Greenhouse-Geisser correction, *η_G_^2^* = .33. This measure of attentional efficiency showed a consistent improvement over the experiment, exhibited in significant differences between all four Blocks in post hoc analysis. We detected no significant effect of Block *F*(3, 66) = 1.50, *p = *0.22, on TIPSand so there is no evidence of significant change over the course of the experiment. A single sample t-test, *t*(22) = 2.18, *p = *0.04, *d* = 0.45 revealed a significant attention change bias to error trials (*M* = 0.04, *SD* = 0.09). This test indicated that participants are more likely to change their attention patterns following an error trial than after a correct trial.

### Experiment 5. A Simple Case of Information Integration: A Two-category Experiment

**Figure 5:**
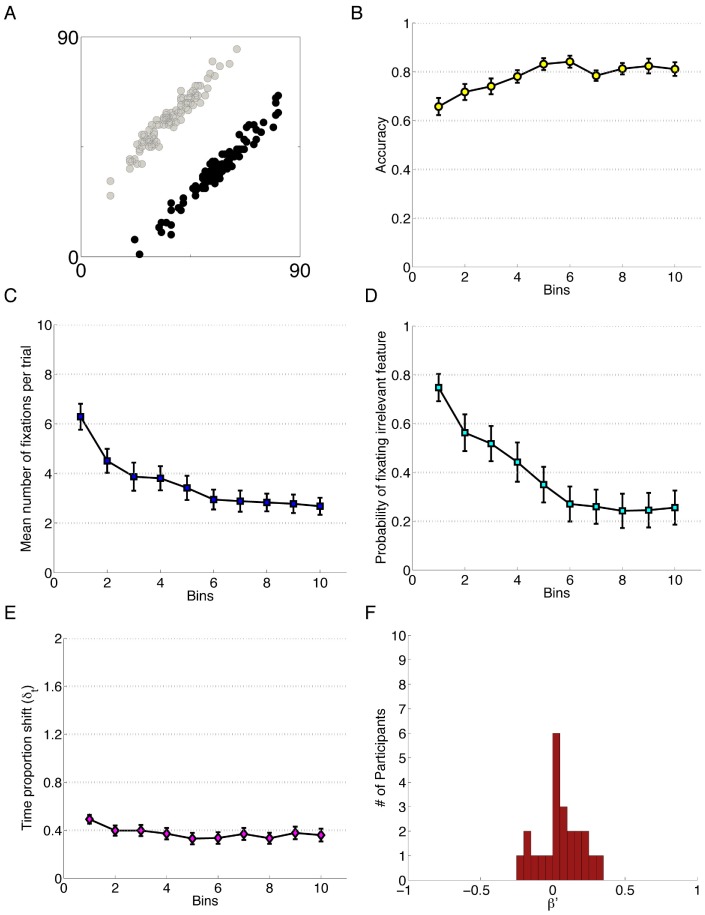
Data for Experiment 5. Each bin reflects the average of 20 trials and error bars represent standard error of the mean. A) The category structure. B) Performance accuracy. C) Mean number of fixations per trial. D) Probability of fixating the irrelevant feature. E) Time proportion shift, δ_t_. F) Error Bias, β′.

The information integration structure used in Experiment 4 created a relatively challenging categorization task. Here we explored a simpler case of information integration in Experiment 5, followed by a simpler case of rule based learning in Experiment 6. The category structure was similar in design to Experiments 3 and 4, in that stimuli were defined by continuously valued features with a linear bivariate dependency (see Blair *et al*, [31]). The difference in this experiment was that there are only two categories (Figure 5A), rather than the four that were learned in the previous experiments. The data for Experiments 5 and 6 can be accessed online through SFU Summit under the title “2 Category Continuous Dimension Learning”.

Figure 5A represents the two-category information integration task used to produce the stimuli in this experiment. There was a third stimulus dimension that was not diagnostic of the correct category. This kind of category has seen widespread use in the category learning literature (e.g. [29], [30]). Other analyses using these data were published in a report by Blair, Chen, Meier, Wood, Watson, and Wong [31] where methodological details and the precise distributional parameters for the stimulus features were described. Technical specifications of the stimulus presentation and gaze collection were identical to Experiments 3 and 4. Fifteen participants failed to respond correctly over 13 consecutive trials and were excluded, leaving 23 participants in the analysis, all of whom met the gaze criteria.

#### Results

Again, a series of analyses were conducted on the measures of interest, with each level of Block reflected the average of 50 trials. These data are visualized in Figure 5, and the means and standard deviations are shown in Table 5 in Text S1, “Four Block Experiment Data”. A linear effects model predicting response accuracy data from this experiment showed that Block was an important predictor of accuracy, β_block_ = 0.23, *z* = 6.63, *p*<0.001. A second linear mixed effect model using Block to predict the probability of fixating irrelevant information showed that it is a significant predictor, β_block_ = −1.14, *z* = −21.38, *p*<0.001. An analysis of the number of fixations is conducted, with Block as a factor in an ANOVA, and we found a main effect of Block *F*(2.56, 56.23) = 42.11, *p*<0.001 after Huynh-Feldt correction, *η_G_^2^* = 0.18. Post-hoc analysis revealed significant differences between all Blocks except for Block 3 and 4. However, we detected no significant effect of Block on TIPS, *F*(3,66) = 1.69, *p = *0.18.

An interesting complexity arises in cases like this, since efficiency was improving over the course of the experiment but generally there were high levels of attention change. This may be due in part to the relative ease of the task - given that there were only two categories it took less time to sort out an appropriate information access strategy and the attention change measure hit a floor value very early on. Another possibility is that participants were relying on an implicit learning system, and their knowledge of the category was tied very closely to learned oculomotor movements rather than to abstracted, verbalizable rules, which would be consistent with the claims made by Maddox and Ashby [32].

A single sample t-test, *t*(22) = 1.77, *p* = 0.09, *d* = .037 revealed a marginally significant Error Bias, (*M = *0.05, *SD = *0.14).

### Experiment 6. Single Dimension Rule Use: A Simpler Two-category Experiment

**Figure 6:**
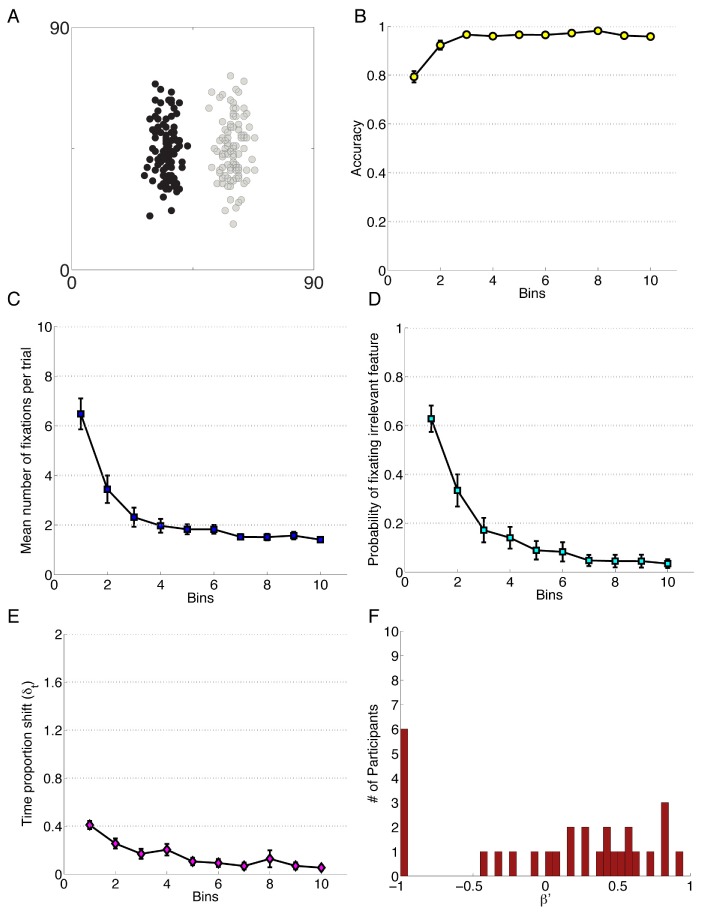
Data for Experiment 6. Each bin reflects the average of 20 trials and error bars represent standard error of the mean. A) The category structure. B) Performance accuracy. C) Mean number of fixations per trial. D) Probability of fixating the irrelevant feature. E) Time proportion shift, δ_t_. F) Error Bias, β′.

As with Experiment 5, the categories in this experiment were determined by a single boundary in a continuous feature space. However, only one of the three features was relevant for classification (see Figure 6A). The two-category, single-dimensional, rule-based category structure was similar to that used by Maddox and Ashby [32] and other studies, but instead had spatially-separated features. Due to the simplicity of this task relative to the others, we expected higher accuracy, fewer fixations per trial, decreased probability of fixating the irrelevant feature, a rapid decrease in TIPS and an Error Bias.

#### Participants

Thirty-three undergraduates received partial course credit for their participation in the experiment. Two participants failed to reach the learning criterion of 13 consecutive correct trials and were excluded from analysis, leaving 31 participants, who all met the gaze criteria, in the analysis.

#### Stimuli and category structure

Figure 6A shows the category structure learned by the participants in this experiment. Like the other structures, the stimuli were three dimensional, but unlike the other structures only one of these features was diagnostic of category membership. Additional analyses using these data were published in Blair, Chen, and colleagues [31].

#### Procedure

The procedure was identical to that used in Experiment 5 with the eye-tracker sampling at 50 Hz; here again, participants are presented with 200 trials in a category learning task.

#### Results

In these analyses, Block reflects the average of 50 trials. Data are visualized such that each bin reflects 20 trials in Figure 6, the means and the standard deviations for which are in Table 6 in Text S1, “Four Block Experiment Data”. A linear mixed effects model created to predict accuracy shows that Block is an significant predictor of accuracy, β_block_ = 0.56, *z* = 9.53, *p*<0.001. In a second model, we saw that Block is also an important predictor of the probability of fixating irrelevant information, β_block_ = −1.45, *z* = −28.16, *p*<0.001. The four levels of Block acted as a within subjects factor to analyze the number of fixations over the course of the experiment using an ANOVA. There was again a main effect of the Block on the number of fixations, *F*(1.27, 38.00) = 40.33, *p*<0.001, *η_G_^2^* = 0.34. Post hoc analysis revealed a significant difference between Block 1 and the rest of the Blocks, as well as between Block 2 and 4.The final ANOVA was conducted on TIPS, excluding a single participant who had a cell of missing data. There was a main effect of Block on TIPS, *F*(2.67, 77.55) = 15.38, *p<*0.001 after Huynh-Feldt correction, *η_G_^2^* = 0.17. Once again, post hoc analyses reflected the same significant differences as the previous two measures, in that there was a difference between Block 1 and all subsequent Blocks, and between Blocks 2 and 4. Finally, the value of Error Bias was tested against a mean of zero to uncover any bias to change attention following error (or correct) trials. Due to substantial violations of normality in the Error Bias distribution, a non-parametric Mann-Whitney U test against a median of 0 was employed rather than a t-test, *Mdn* = 0.28, *M* = 0.07, *SD* = 0.65, *z* = 0.66, *p*<0.51, detecting no significant bias. Similar to Experiments 3 and 4, Experiments 5 and 6 might have encouraged differing explicit and implicit processing strategies. A test of this hypothesis again detected no basis to this claim in our observed Error Bias data using a non-parametric Mann-Whitney U test of unpaired samples, showed *z* = 1.29, *p = *0.197.

### Experiment 7. A Single Dimension Rule with a Speed/accuracy Condition Crossing

The category structure used here is similar to the Type 1 Category reported in the seminal work by Shepard, Hovland and Jenkins [33]. A critical difference here is that the categories were not based on continuously varying features, like in Experiments 3–6, but instead were comprised of features that assumed binary values like Experiments 1 and 2. Like Experiment 2, this experiment contained an instruction manipulation that encouraged participants to be either fast or accurate. The raw data are available in the collection titled “Speed-Accuracy Trade-Offs in Category Learning”, hosted at summit.sfu.ca/collection/94.

#### Participants

Sixty-seven undergraduates participated in this experiment. Three were excluded for poor eye gaze quality, and an additional seven were excluded for failing to reach the learning criterion of 12 consecutive trials, leaving 57 participants for the analysis: 29 in the Speed condition and 28 in the Accuracy condition.

#### Stimuli and category structure

Participants learned two categories in this task. Each of the three features could take one of two possible values. Only one of the features determined the category, while the remaining two features were irrelevant (Table 1). Organelle images are shown in Figure S2, “Stimulus and feature images for Experiments 2 and 7”, for reference.

**Table 1:** The category structure used in Experiment 7.

Category	Feature 1	Feature 2	Feature 3
A	0	0 or 1	1 or 0
B	1	1 or 0	0 or 1

#### Procedure

**Figure 7:**
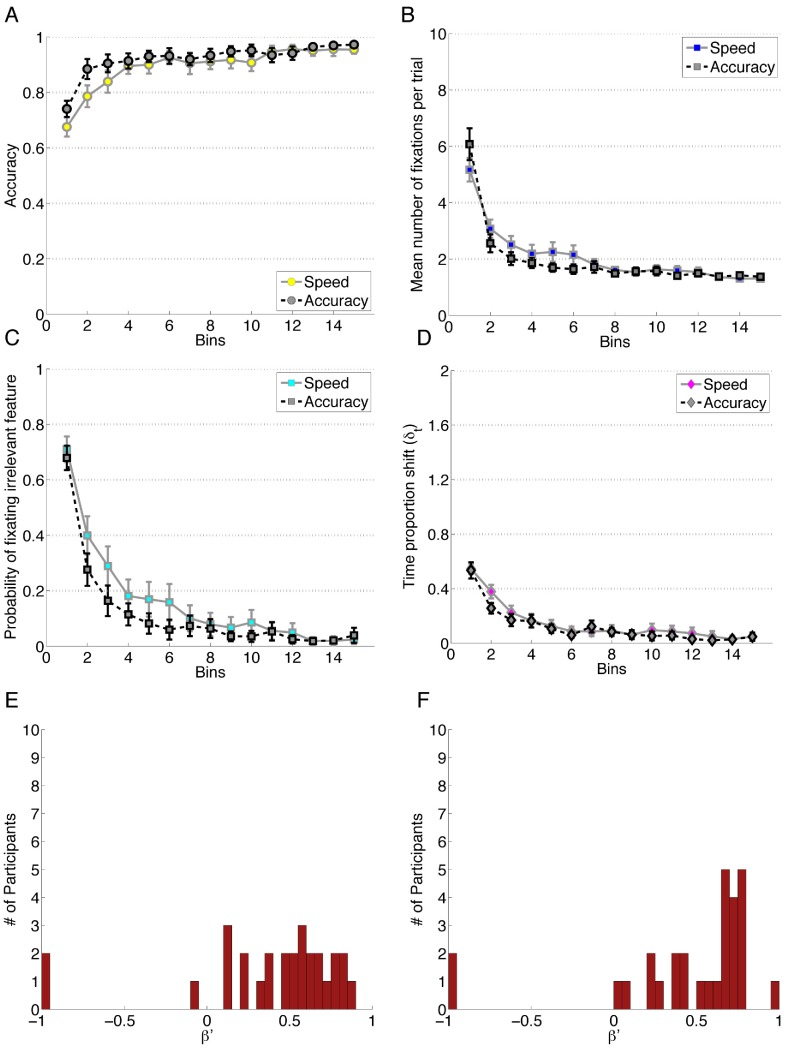
Data for Experiment 7. Each bin reflects the average of 20 trials and error bars represent standard error of the mean. A)Performance accuracy. B) Mean number of fixations per trial. C) Probability of fixating the irrelevant feature. D) Time proportion shift, δ_t_. E) Error Bias, β′, for the speed condition. F) Error Bias, β′, for the accuracy condition.

The experiment consisted of 300 trials in 15 blocks of 20. On each trial, participants saw the stimulus and chose a category response from the two possible categories. Responses were made on a Logitech game pad’s trigger buttons in the same manner as Experiment 2. Following the response, the participant’s selected button was highlighted in red or green to communicate the trial accuracy and the stimulus was simultaneously re-presented. All gaze collection and stimulus specifications were the same as those detailed in Experiment 2. Each Block represents the aggregate of 75 trials or 3.75 bins of Figure 7.

#### Results

Data are visualized in Figure 7. A linear mixed effect model is constructed to predict the accuracy of participants using Block and Condition as predictors. Block is a significant predictor of accuracy, β_block_ = 1.05, *z* = 10.122, *p*<0.001 as are Condition, β_block_ = 1.63, *z* = 5.05, *p*<0.001 and the interaction between the two, β_block_ = −0.53, *z* = −3.62, *p*<0.001. A linear mixed effects model showed that Block is a significant predictor, β_block_ = −2.36, *z* = 10.424, *p*<0.001, as was Condition, β_condition_ = −2.50, *z* = 3.94, *p*<0.001. The interaction term was not a significant predictor of the probability of fixating irrelevant information β_block × condition_ = −0.05, *z = *−0.15, *p = *0.88. An ANOVA was conducted on the number of fixations to features. Again, we found a significant main effect of Block, *F*(1.75, 96.30) = 46.56, *p*<0.001 after Greenhouse-Geisser correction, *η_G_^2^* = .23. There was no main effect of the Instruction Condition, *F*(1,55) = 0.27, *p* = 0.60 and no interaction between the factors, *F*(1.75, 96.30) = 0.87, *p* = 0.41. There were significant differences between all four Blocks via post-hoc analysis on the collapsed data. The same factors were used to analyze the TIme Proportion Shift (TIPS) where one subject was excluded from analysis for one cell of missing data. We found a significant main effect of Block, *F*(2.43,131.11) = 41.88, *p*<0.001 after Huynh-Feldt correction, *η_G_^2^* = 0.20 with no significant main effect of Instruction Condition, *F*(1,54) = 0.32, *p = *0.57 or interaction, *F*(2.43,131.11) = 0.35, *p = *0.75. Post hoc analysis revealed a significant difference between Block 1 and all other Blocks, as well as Block 2 and 4.

To determine if the speed/accuracy manipulation influences the Error Bias, a Mann Whitney U test of unpaired samples was performed as there were substantial violations of normality in the samples. The result of this test showed no detectable differences between condition, *z = *−1.00, *p* = 0.318. A non-parametric Mann Whitney U test against a median of 0 was performed for the combined speed and accuracy conditions, *Mdn* = 0.57, *z* = 4.78, *p*<0.001, *r* = 0.63, revealing a significant attention change bias towards error responses.

### Experiment 8. A Complex Contingency Category Learning Task

**Figure 8:**
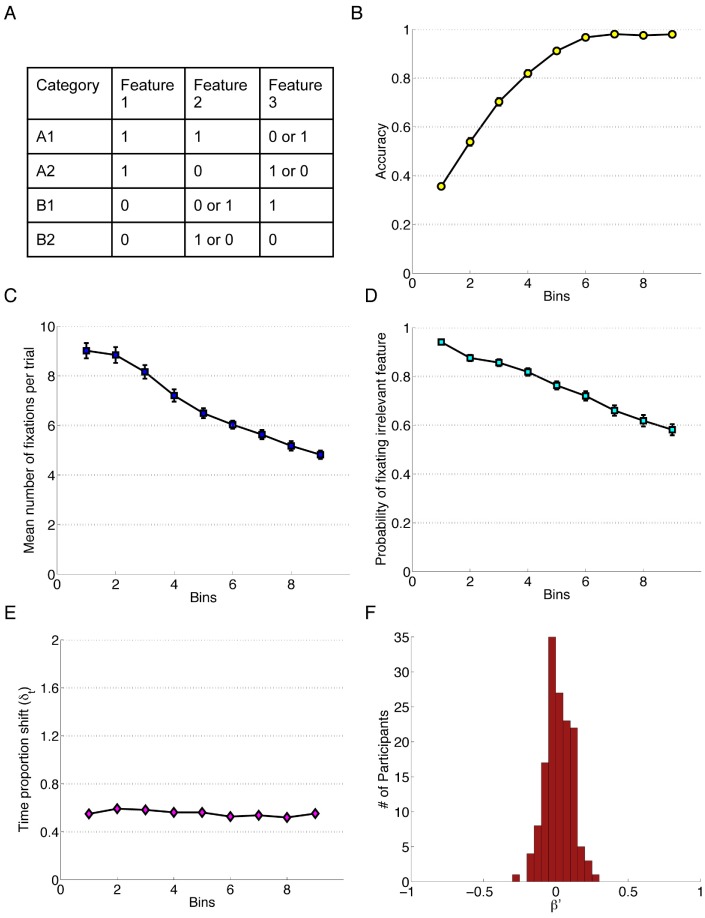
Data for Experiment 8. Each bin reflects the average of approximately 20 trials depending on how quickly each individual learned the categories Error bars represent standard error of the mean. A) The category structure. B) Performance accuracy. C) Mean number of fixations per trial. D) Probability of fixating the irrelevant feature. E) Time proportion shift. F) Error Bias, β′.

In contrast to the earlier experiments, the relevant dimensions in this task were *stimulus specific,* meaning that each category yielded its own optimal attention pattern [18]. This experiment examined the influence of having the information necessary for optimally completing each trial embedded in the trial itself, rather than learned spatial patterns that can be deployed constantly in each trial in the experiment like the tasks above. Attentional optimization was more difficult in this experiment, and participants continued to slowly optimize attention well after performance errors had ceased. The category structure, found in Figure 8A, was designed to elicit a stimulus specific attentional pattern such that the relevance of Feature 2 and Feature 3 was contingent upon the value of Feature 1; participants can optimize their attentional patterns by looking at Feature 1 first. Features 1 and 2 were relevant for two categories (A1 and A2), and Features 1 and 3 are relevant for the other two (B1 and B2). The feature location relevance was counterbalanced among participants.

The length of the experiment varied among participants. If the learning criterion, 25 correct trials in a row, was reached prior to the 200th trial than the participant would only have to do 72 more trials. These final 72 trials did not contain performance feedback. Otherwise, the experiment lasted a maximum of 272 trials but those who failed to learn by the 200th trial are excluded from the following analyses and identified as non-learners. Additional methodological details and technical specifications are available in Chen and colleagues’ work [22], which report a different set of results than those discussed below. The data can be accessed at summit.sfu.ca/collection/94 under “Stimulus Specific Association with Learning Dependent Feedback”.

#### Results

It is important to note that, unlike in all other experiments, the number of trials in this experiment varied across participants. The end of the experiment was determined by how quickly participants learned the categories, and so the actual number of trials in each plot bin of Figure 8, and in each Block of the analysis, may differ slightly between participants. Like in previous experiments, the data to be included in Figure 8 bins were calculated by the proportion of the experiment total, such that the bin size was approximately 20 trials to align with the other experiments. Although there was a slight difference in the number of trials, the data included in the blocks are more sensitive to participants’ place along the learning trajectory. Namely, the mastery of the task for those who learn quickly was compared closely with those who learn slowly in that the learning curves were dependent on when exactly participants achieved the learning criterion. In these analyses, Block (1–4) reflects an average of 46 trials per block. A linear mixed effects model was constructed to predict participants’ response accuracy using the Block number, and revealed that Block was an effective predictor of accuracy, β_block_ = 1.54, *z* = 63.33, *p*<0.001. The probability of fixating irrelevant information was predicted using a linear mixed effects model with Block as a significant predictor, β_block_ = −0.68, *z* = −41.62, *p*<0.001. There was also a main effect of Block on the number of fixations, *F*(1.82, 263.84) = 140.34, *p<*0.001 after Greenhouse-Geisser correction, *η_G_^2^* = .24. Post hoc tests showed a significant difference between all Blocks, which suggested that the number of fixations continued to decrease over the course of the whole experiment. A main effect of Block on TIPS is found too, *F*(2.58, 374.33) = 4.21, *p = *0.009 after Huynh-Feldt correction, *η_G_^2^* = 0.01. Post-hoc analysis revealed the only significant mean difference was between Blocks 1 and 3. As one can see from the Figure 8F, a very slight Error Bias was detected (*M* = 0.02, *SD* = 0.09). A one-sample t-test confirmed that the sample mean was significantly different from zero *t*(145) = 2.64, *p = *0.009, *d* = 0.22.

### Experiment 9. Efficiency and Accuracy Manipulation over Extended Time

This experiment was conducted to investigate how participants solving a structure akin to that of Experiment 8 would change their attentional patterns if the frequency of the categories they saw was altered such that the informative value of category features changed. Methodological details and technical specifications are available in Meier and Blair [34] who report a different set of findings. Like Experiment 8, this task uses a category structure that was designed to invoke stimulus specific attention (Table 2). Participants were assigned to one of two possible conditions: the 1∶1 ratio condition wherein all categories were equally probable; or the 5∶1 condition wherein two of the four categories were presented five times as often. The data can be found at summit.sfu.ca/collection/94 under the title “Probability Gain versus Information Gain in Category Learning: Eye Movements”.

**Table 2:** Category structure for stimuli in Experiment 9.

Category	Feature 1	Feature 2	Feature 3	5∶1 ConditionCategory Frequency	1∶1 ConditionCategory Frequency
A1	0	0	0 or 1	5/12	1/4
A2	0	1	1 or 0	5/12	1/4
B1	1	0 or 1	0	1/12	1/4
B2	1	1 or 0	1	1/12	1/4

#### Results

**Figure 9:**
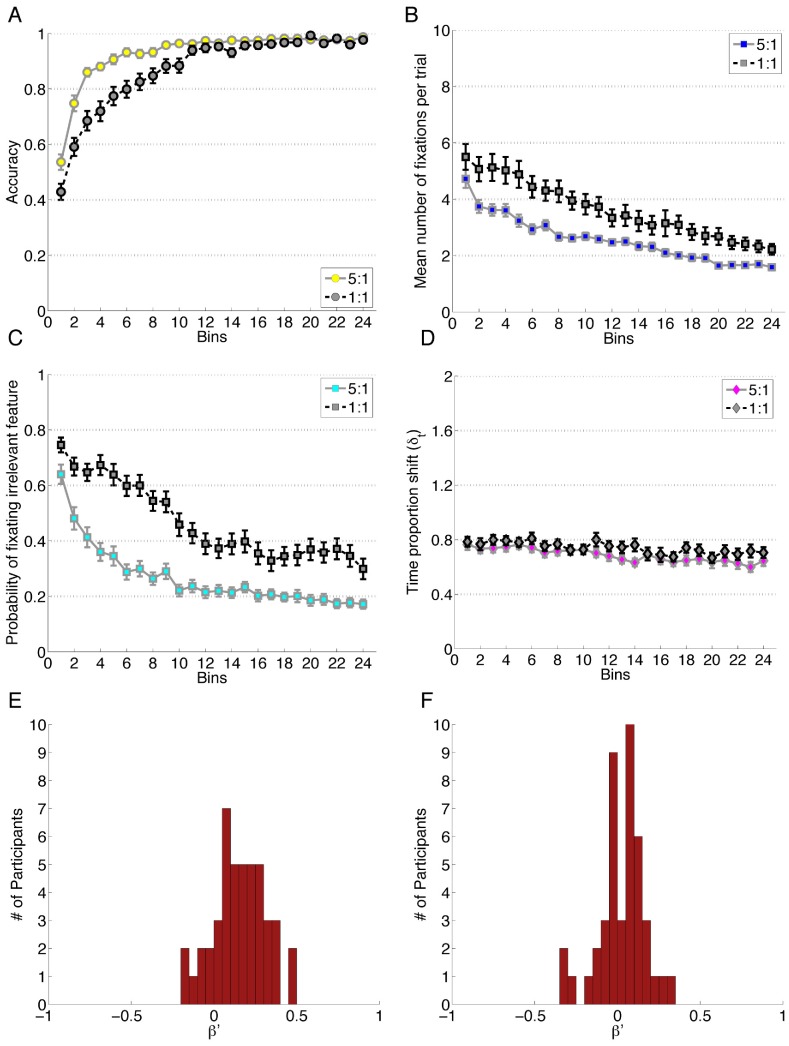
Data for Experiment 9. Each bin reflects the average of 20 trials and error bars represent standard error of the mean. A) Performance accuracy. B) Mean number of fixations per trial. C) Probability of fixating the irrelevant feature. D) Time proportion shift, δ_t_. E) Error Bias, β′ for the 5∶1 condition and F) for the 1∶1 condition.

Analyses are conducted using the between-subjects factor Probability Condition (1∶1, 5∶1) and the within-subjects factor Block (1–4). In this analysis, each Block contains 120 trials, or 6 bins of Figure 9. Accuracy scores were predicted using a linear mixed effects model, which included Block, Condition and the interaction between them as predictors. The model shows that Block, β_block_ = 0.97, *z* = 11.90, *p*<0.001, Condition, β_condition_ = −0.72, *z* = −3.993, *p*<0.001, and the interaction, β_block × condition_ = 0.34, *z* = 2.85, *p* = 0.004 were all important predictors of accuracy. The increased learning speed in the 5∶1 condition is thought to be a result of learning the high presentation categories quickly and using that knowledge to bootstrap their learning for the remaining categories.

The probability of fixating irrelevant information was predicted by a linear mixed effects model, which showed Block, β_block_ = −0.37, *z* = −7.27, *p*<0.001, Condition, β_condition_ = 1.05, *z* = 6.65, *p*<0.001 and the interaction between Block and Condition, β_block × condition_ = −0.16, *z* = 2.05, *p* = 0.04 were all significant predictors. An ANOVA conducted on the number of fixations per trial revealed a significant main effect of Condition, *F*(1,87) = 12.70, p<.001, *η_G_^2^* = .10, such that participants in the 5∶1 Condition fixated fewer features; a significant main effect of Block, *F*(1.62, 140.71) = 36.85, *p*<.001 after Greenhouse-Geisser correction, *η_G_^2^* = .10; and no interaction between the two, *F*(1.62, 140.71) = 1.87, *p = 0.*17. Post-hoc analyses on Block revealed that each level of Block was significantly different from each other level.

Analyses on attention change, measured by TIPS detects no main effect of Condition, *F*(1, 87) = 3.73, *p = *0.057. There was however an effect of Block, *F*(2.72, 236.64) = 9.80, *p*<0.001 after Huynh-Feldt correction, *η_G_^2^* = .03 but no detected interaction between Condition and Block, *F*(2.72, 236.64) = 0.23 after Huynh-Feldt correction, *p = *0.87. A post-hoc follow up shows significant differences between all Block pairs except for Blocks 1 and 2 and between Blocks 3 and 4.

An independent samples t-test of the Error Bias distributions, *t*(87) = 4.13, *p*<0.001, *d = *0.88, SD = 0.15, showed that an unequal presentation rate of categories yielded more Error Biased eye-movements. Each distribution was tested separately as a result in a single sample t-test. When the categories are all equally likely (1∶1), a t-test against a mean of 0, *t*(43) = 1.43, *p* = 0.16, detected no effect of error on attention change (*M* = 0.03, *SD* = 0.13). However, a similar t-test for the 5∶1 condition, *t*(44) = 6.72, *p*<0.001, *d = *1.00, revealed a significant Error Bias (*M* = 0.16, *SD* = 0.16).

Visual inspection of Figures 8 and 9 indicated that there were differences in the 1∶1 condition in Experiment 9 (Figure 9), and Experiment 8 (Figure 8) which would be unexpected at first pass given that the category rule in Experiment 8 and the 1∶1 condition of Experiment 9 are similar. An important difference in the visualization is the manner in which the bins are formed, where the final trial in Experiment 8 is determined by how quickly participants learned. For instance, someone who learned the task early may have their 70^th^ trial in the final bin, whereas someone who learned much later may have their 70^th^ trial in the third bin. The final trial in Experiment 9 is always the 480^th^ trial regardless of participants’ performance. The disparity between the measures was largest in the first bin, and lessens in the 9th bin, suggesting that part of the difference was a function of learning-contingent end points used for Experiment 8. There were important differences to note between the two experiments in themselves, however. In Experiment 8, the stimulus was not on screen during the collection of the category response. This results in a heavier cognitive load, since participants made their category decision while the stimulus was on screen, then memorized the appropriate label and then used that label to make their response using the scrambled response buttons on the next screen. Another critical difference between the two was that each trial in Experiment 8 begins with a central fixation cross that had to be clicked before moving on to stimulus presentation, whereas in Experiment 9 the central cross did not require a directed manual movement. The contributions of these small differences between Experiment 8 and 9 require further investigation, but regardless of the weighting of the task differences in eliciting the varying results, we take the changes in these measures as evidence for their sensitivity to task variation and as support for their efficacy in capturing subtle differences in cognitive processing.

### Experiment 10: Hand-based Information Access

This experiment extends the findings elicited by the category structure used in Experiment 9. Additional analysis from this experiment, not reported here, are included elsewhere where the methods and materials are also described [34], and the raw data are found through SFU summit, collection 94 entitled “Probability Gain versus Information Gain in Category Learning: Hand Movements”. The critical difference between this experiment and the ones that precede it was the manner in which participants access information. Instead of having only to make eye movements to gather the information from each of the features, participants used mouse movements to uncover features that were obscured by visual noise masks. Approximately half of the participants were assigned to a condition with a three-second delay between the time a feature was moused over, and when a feature was revealed. This manipulation was designed to probe how strategies geared towards maximizing either efficiency or accuracy was mediated by access cost (i.e. the delay in uncovering the feature). The stimuli were the same as in the previous experiment, but in this experiment they were covered with a mask so participants could not see the feature itself until they hovered over the mask with a mouse cursor. In the no delay condition, the feature was shown immediately, and in the delay condition the participant had to keep the mouse cursor over the mask for three seconds to see the feature underneath it.

#### Results

**Figure 10:**
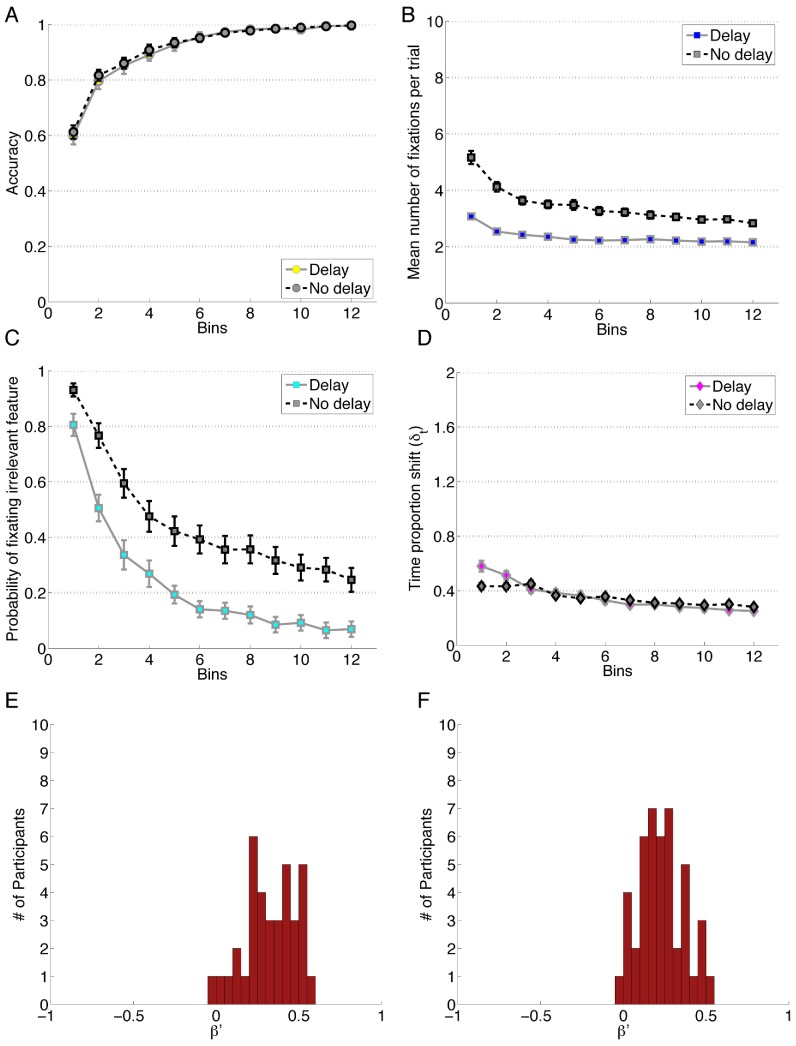
Data for Experiment 10. A) Performance accuracy. B) Mean number of fixations per trial. C) Probability of fixating the irrelevant feature. D) Time proportion shift, δ_t_. E) Error Bias, β′, for the delay condition and F) for the no delay condition. Each bin reflects the average of 20 trials.

The conditional means and standard deviations for all of the measures, along with details of the linear mixed effects models reported here are available in Table 10 in Text S1, “Four Block Experiment Data”. For this experiment there were 60 trials per block, 3 bins of Figure 10. A linear mixed effects model was constructed to predict accuracy using Block, Delay Condition and the interaction between the two. The model showed Block to be a significant predictor, β_block_, 1.79, *z* = 14.73, *p*<0.01; but revealed that Condition, β_condition_ = −0.05, *z* = −0.034, *p* = 0.738, and the interaction between Condition and Block, β_block × condition_ = 0.09, *z* = 0.51, *p* = 0.61, were not significant predictors.

A linear mixed effects model designed to predict the probability of fixating irrelevant information showed that Block, β_block_ = −1.03, *z* = −7.649, *p*<0.001, Condition, β_condition_ = −1.38, *z* = −3.18, *p*<0.001 and the interaction of the two, β_block × condition_ = −0.50, *z* = −2.471, *p* = 0.013 were all important predictors. Analyses of the number of fixations to features showed that there was a significant main effect of Condition *F*(1, 80) = 74.43, *p*<0.001, *η_G_^2^* = .40, and there was a significant effect of Block *F*(1.87, 149.48) = 10.76, *p*<0.001 after Greenhouse-Geisser correction, *η_G_^2^* = .04. There was also a significant interaction between Condition and Block, *F*(1.87, 149.48) = 17.04, *p*<0.001 after Greenhouse-Geisser correction, *η_G_^2^* = .06. Follow-up analyses revealed a significant simple main effect of Block in both the delay condition, *F*(1.87, 149.48) = 10.76, *p*<0.001, and in the No-delay condition, *F*(1.87, 149.48) = 93.69, *p*<0.001. Post-hoc analyses showed significant difference between the first block and all other blocks, as well as a significant difference between Blocks 3 and 4 for the delay condition, and significant difference in all block pairs in the No-delay condition.

The TIPS was analyzed with a mixed model ANOVA wherein no effect of Condition was found *F*(1,80) = 0.01, *p = *0.91. No main effect of Block *F*(2.18, 174.60) = 55.26, *p*<0.001 after Greenhouse-Geisser correction, *η_G_^2^* = 0.22 was detected but there was an interaction between Condition and Block, *F*(2.18, 174.60) = 4.67, *p = *0.008, *η_G_^2^* = 0.02. Follow-up analyses showed significant effects of Block for the delay condition, F(2.18, 174.60) = 55.26, *p*<0.001, and for the no delay condition, F(2.18, 174.60) = 27.00, *p*<0.001. Post-hoc followups showed a difference between all Block pairs, except between Blocks 2 and 3 in the delay condition.

An independent samples t-test, *t*(80) = 2.91, *p* = 0.005, *d* = 0.65, *SD = *0.14 showed the two Error Bias distributions differ. In the delay condition, a single sample t-test, *t*(36) = 12.97, *p*<0.001, *d* = 2.13, revealed a significant attention change bias on error (*M = *0.33, *SD = *0.15). In the no delay condition, a single sample t-test, *t*(44) = 11.39, *p*<0.001, *d* = 1.70, again revealed a significant attention change bias towards error responses (*M = *0.23, *SD = *0.14) but with a smaller effect size. Notably, these results replicate the eye-movement Error Bias scores for the similar unbalanced category presentation condition (5∶1) of Experiment 9, which was also shown to be positive.

## General Discussion

This dataset from nearly 600 participants, spanning ten experiments, presents a unique contribution to attentional learning research in several different ways. Because of the complexity of human attention and the relative paucity of data on the relationship between overt attention and category learning, advancement of the field will require more data than is usually presented for scientific publication. The data provided here cement the kinds of patterns and variation that can be expected from eye-movements during learning, as they are both methodologically sound and congruent with previous research. For instance, as accuracy improves, the number of fixations made during a trial decreases, and those fixations are deployed more efficiently [17], [21]. This is as much of a litmus test as can currently be constructed for eye-tracking data during categorization because of how sparse the established findings are. With respect to the utility of the novel measures, TIPS and Error Bias, they each exhibit desirable properties in that they respond to subtle changes in the task, but are also quite consistent in important ways - TIPS generally, though not always, tends to decrease over the experiment and Error Bias is often detected, but often with small to medium effect sizes, reflecting no strong bias to change attention following error or correct trials. Further, these measures can be related back to the more standard measures, like the accuracy and optimization, in ways that make sense and are available for modeling at a finer grained, nested individual level. In order to better extract the theme that emerges for each measure over the course of the experiments, and how those themes relate to one another, it is important to look at each measure individually.

### Accuracy

As has been established in previous work [21], attention is, predictably, influenced by a participants’ knowledge of the category structure. We see this consistent trend for accuracy to improve over the course of the experiment, with variation that confirms the expected or intended effect of a particular experimental manipulation. For example, the accuracy for a single dimensional rule task is higher relative to other category structures over similar trials. Conversely, accuracy tends to be lower when categories are based on continuous dimensions, which is thought to be a function of the more difficult perceptual discrimination underlying the category choice. In many ways the accuracy data are unsurprising. However, these learning curves have not previously been reported for the given category structures when paired with attention data, so the findings have utility for the modeling community, above and beyond their role in confirming the efficacy of the experimental manipulations.

### Probability of Fixating Irrelevant Information

All category structures reported in this paper have at least one irrelevant feature. The probability of fixating irrelevant information acts as a measure of attentional efficiency in the task, since a highly efficient participant will dedicate their effort to fixating the relevant feature(s) and ignoring the information that does not inform their category choice. We find that participants become more efficient with the deployment of their attention over the course of a task. Generally, participants become highly efficient after their accuracy begins to asymptote. This finding serves both to replicate earlier work [17] and to pair externally verified measures with the ones we introduce for the first time in this paper.

### Number of Fixations

The number of fixations measures attentional efficiency in a different way: rather than testing where the participants’ fixations are deployed, this measure tests simply how many fixations are deployed to features in total. Efficient participants will minimize the number of fixations they make prior to responding with their category choice, since the information on the stimulus remains constant for the duration of the trial and there is no need to revisit information that has already been fixated and encoded, especially if the information is unimportant for the category decision. The findings here replicate those previously reported and allow for interesting pairings such as how the number of fixations might relate to the TIPS measure as a function of sample size and the central limit theorem (i.e. as there are more fixations, the measures here should approximate a normal distribution and the proportion of time spent on each feature will be more robust to the possibility of erroneous sampling that may mask changes in the allocation of attention trial-to-trial). Unfortunately, it is hard to draw inferences from the findings here on this possibility.

### Time Proportion Shifts (TIPS), δ_t_

TIPS was developed to measure changes in trial-to-trial attentional allocation. The theoretical motivation for this measure arises, in part, from formal models of attentional change in categorization that predict attentional patterns should stabilize over the course of learning. The rationale for decreasing attentional changes over learning is that once the system has found an ideal response pattern then attention change is no longer necessary for improving performance [15]. We find the predicted decrease in attentional changes through TIPS in Experiments 1, 2, 6, and 7, for which there is a main effect of Block. score. The remaining experiments fail to elicit a detectable change in TIPS over the course of the experiment.

There are a number of factors varying between the experiments that evidence attentional change over learning versus those that do not. Tasks built on category structures such as the ones in Experiments 6 and 7 are defined by simple one dimensional rules. Tasks with more complex perceptual discrimination (e.g. Experiments 3, 4) or more complex rules for categorization (e.g. Experiments 8, 9 and 10) do not yield a significant TIPS score. Both task complexity and the difficulty of perceptually discriminating stimulus features may play a role in the change of attention over learning, but additional experiments will be needed to establish their influence.

A possible limitation in comparing the attention change data from Experiment 9 and 10 is that eye-movements differ from hand movements how they’re used during and after learning [35], although they are often correlated in goal directed activity. The relationship between the two has been previously formalized by measures like eye-hand span [36], which captures the direction and latency between the two movements. This kind of measure has been used to study complex motor movements, such as speed stacking cups, (a unique and complex sensorimotor task capable of being quickly learned) and in novel tool use. It is generally found that over the course of the development of expertise in tasks that require joint coordination between the eyes and hands, that eye movements will begin to precede hand movements to task relevant objects. This points to a dynamic relationship between the two movements, with shifting roles and specializations. Although mouse movements, like those in Experiment 10, require less energy than rotating or grasping objects, they require more motor resources than eye movements, and consequently, less shifting should be expected [34]. It is not surprising, then, that we see those kinds of qualitative differences at an aggregate level in the TIPS score between Experiments 9 and 10 (Fig. 9F and Fig. 10F).

### Error Bias, β′

The purpose of the Error Bias (β′) score is to measure the tendency for attention change to follow error trials, where β′ = 1 means that all attention change follows error and β′ = −1 means all attention change follows correct trials. Error Biases were primarily clustered around zero, but showed variation that is interesting and requires some explanation. One hypothesis for our pattern of Error Bias findings is that the ease of deploying attention to relevant stimulus dimensions is important. It is necessarily the case in a learning task that error trials occur more frequently at the beginning of the experiment and correspond to the highest amount of attention change in these easier tasks (see Figure 6B and 6E for an example). This observation might lend itself to a sensible critique: the existence of an Error Bias is solely related to the likelihood of error trials being clustered earlier in learning. However, we note cases where attention change is constant throughout the experiment, but an Error Bias is observed (Experiments 3 and 9). These examples show that Error Bias is able to pick up on subtle, aggregate tendencies to shift attention after error that would not be expected by simple comparison of learning curve and TIPS magnitudes. That is, Error Bias is a measure that is meaningful for more than simply aggregating the uncertainty that defines participants in early learning. This is a new measure – one in which the contribution of aggregate accuracy, experiment length, fixation durations, probability matching [37] and other aspects of attention and learning, warrant exploration in future research to determine more completely the mediating relationships of Error Bias. It is for the multitude of additional analyses like this that we have posted all of our data online, down to the level of raw gaze for individuals, but also refined into fixations to reduce that barrier.

Although it is worth considering Error Bias to capture the subtleties described above, its value also lies in minimizing the importance of error that has been so strongly championed by error-driven learning algorithms. In the strict sense, an error driven-learning algorithm should have nearly all of its attention change following error trials such that β′*≅*1: a trend that is not evident, given the effect sizes we see, in the dataset presented here. Although error-driven accounts seem applicable in certain instances [38], we suggest that models based solely on error-driven shifts of attention are not realistic representations of overt attentional changes in human learning.

### Summary

Correctly identifying consistent influences of selective attention in learning has broad implications. One of the key features of selective attention is in its ability to reduce the complexity of an information source by biasing the selection of relevant information. Understanding the factors that tune such behavior has obvious theoretical import in areas like skill training and computer modeling. It has been shown in previous work, such as that by Haider and Frensch [39], that performance on a wide variety of tasks is augmented when processing is limited to task-relevant properties. Research has also implicated impaired ability to selectively attend in a variety of clinical contexts where, for instance, distractibility of autistic children is related to their inability to properly selectively attend to stimuli with certain dynamic properties [40]. With respect to learning, Blair and Homa [41] demonstrated how training with incomplete sources of information can lead to selective attention patterns which hinder performance relative to participants with no prior training at all. These studies highlight the importance of developing models that accurately capture changes in how selective attention is deployed over time. To this end, the between-trial attentional variation highlighted by δ_t_ and β′, indicate that a different kind of formalism, perhaps more embodied, is needed to relate learning and attention.

Work on attentional learning is in its early stages, and the previously small body of empirical data leaves little guidance for researchers interested in modeling this important part of overall learning. By comparison, the study of learned selective attention in the context of categorization has an admirable role model in studies of eye-movements during reading, which has derived many of its findings from its use of very large datasets [42]. Our aim here is to provide a comparable dataset and a basis for a new kind of data-driven theory construction. The inferential claims, that task errors are not major predictors of eye-movement changes and that those changes are relatively stable across learning would appear to be two useful, initial, offerings from this dataset.

## Supporting Information

Figure S1 **Stimulus and feature images for Experiment 1, 9 and 10.** The background (left) is located in the centre of a 1680×1050 resolution display, and the diameter of the background circle is approximately the height of the screen. One value of each of the three features (right) is pasted in the arms of the fictitious microorganism. The features pasted on the background are an example configuration. The three features are span approximately 80×80 pixels. The locations of each type of feature is constant for a single participant during the experiment, but the locations of the features are counterbalanced between subjects.
(TIFF)

Figure S2 **Stimulus and feature images for Experiments 2 and 7.** The background (left) is located in the centre of a 1680×1050 resolution display, and the diameter of the approximate circle surrounding the background image approximately 1000 px. The full display was coloured yellow, like is shown behind the background cell. The one value of each of the three features (right) is pasted in the same locations as the features in Experiments 1, 9 and 10. The three features span approximately 80×80 pixels each.
(TIFF)

Figure S3 **Stimulus and feature images for Experiments 3–6.** The background (left) is located in the centre of a 1680×1050 resolution display, and the diameter of the circle surrounding the background image is approximately 1000 pixels. One value of each of the three features (right) is pasted in the arms of the fictitious microorganism. The three features span approximately 130×130 pixels each, and vary on one dimension over 90 degrees (see Figures 3–6A). The right side of the image shows examples of each of the three features at 0°, 45°, 60°, and 90° of variation of feature value.
(TIFF)

Figure S4 **Stimulus and feature images for Experiment 8.** The background (left) is located in the centre of a 800×600 resolution display, and the diameter of the circle surrounding the background image is approximately 590 pixels. One value of each of the three features is pasted in the arms of the fictitious microorganism. The three features span approximately 80×80 pixels each, and vary on one dimension taking on only two possible values. The left side shows one possible configuration of the features, all with one of the two possible feature values displayed. The location of each type of feature is constant for a single participant during the experiment, but the locations of the features are counterbalanced between subjects.
(TIFF)

Table S1 **The category structures used in Experiments 3 and 4.** For stimulus value tables, *F_1_*, *F_2_*, and *F_3_* denote the stimulus dimension. The physical locations of the dimension on screen and the stimulus image used for the dimension are both counterbalanced.
(DOC)

Table S2 **The category structures used in Experiments 5 and 6.**
(DOC)

Text S1 **Four Block Experiment Data.**
(DOC)
