# Supplementary figures and images for: Learning-Induced Changes in Attentional Allocation during Categorization: A Sizable Catalog of Attention Change as Measured by Eye Movements
Source: PLoS One. 2014 Jan 31;9(1):e83302. doi: 10.1371/journal.pone.0083302 (PMC3908863; doi:10.1371/journal.pone.0083302)

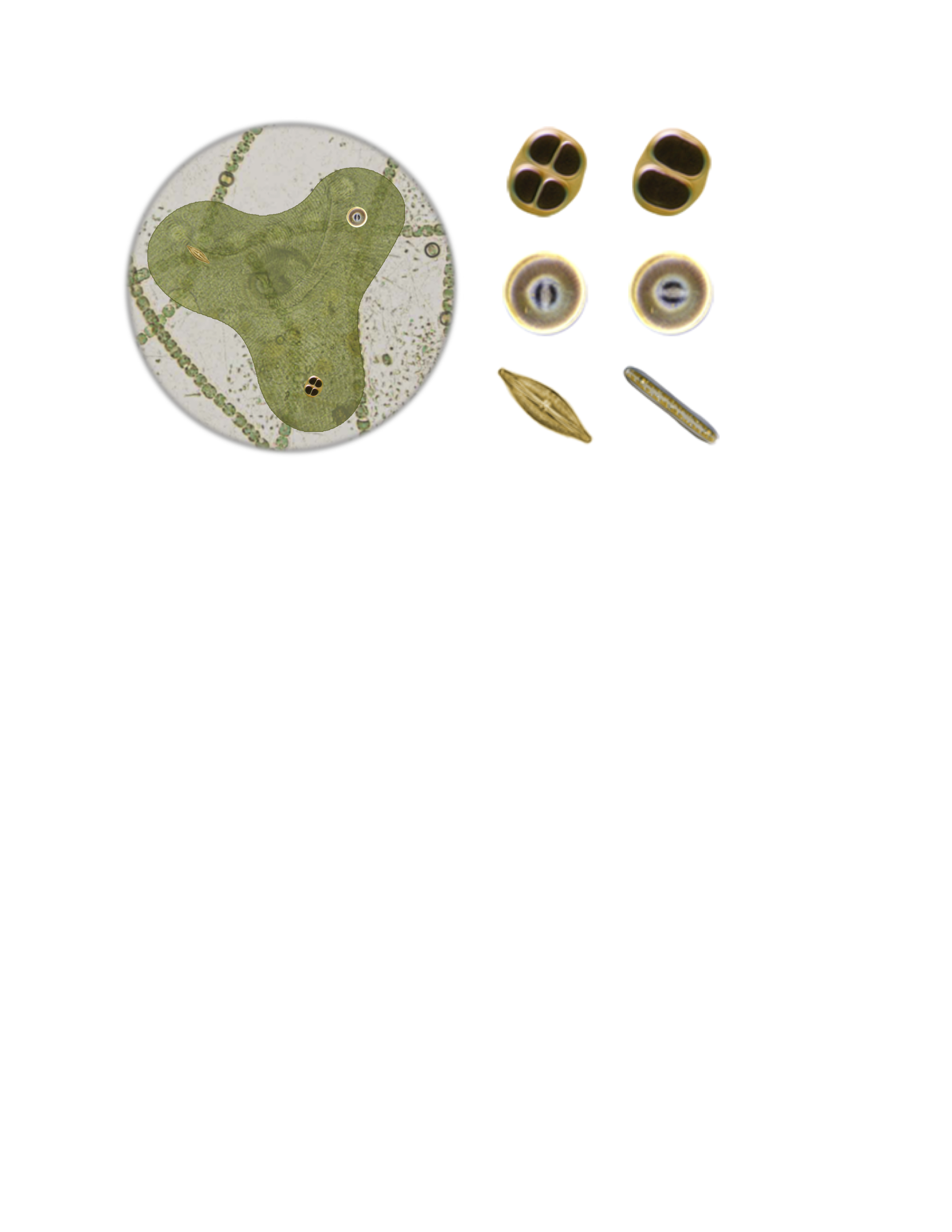

Supplement: Figure S1 — Stimulus and feature images for Experiment 1, 9 and 10. The background (left) is located in the centre of a 1680×1050 resolution display, and the diameter of the background circle is approximately the height of the screen. One value of each of the three features (right) is pasted in the arms of the fictitious microorganism. The features pasted on the background are an example configuration. The three features are span approximately 80×80 pixels. The locations of each type of feature is constant for a single participant during the experiment, but the locations of the features are counterbalanced between subjects. (TIFF) [file pone.0083302.s001.tiff]

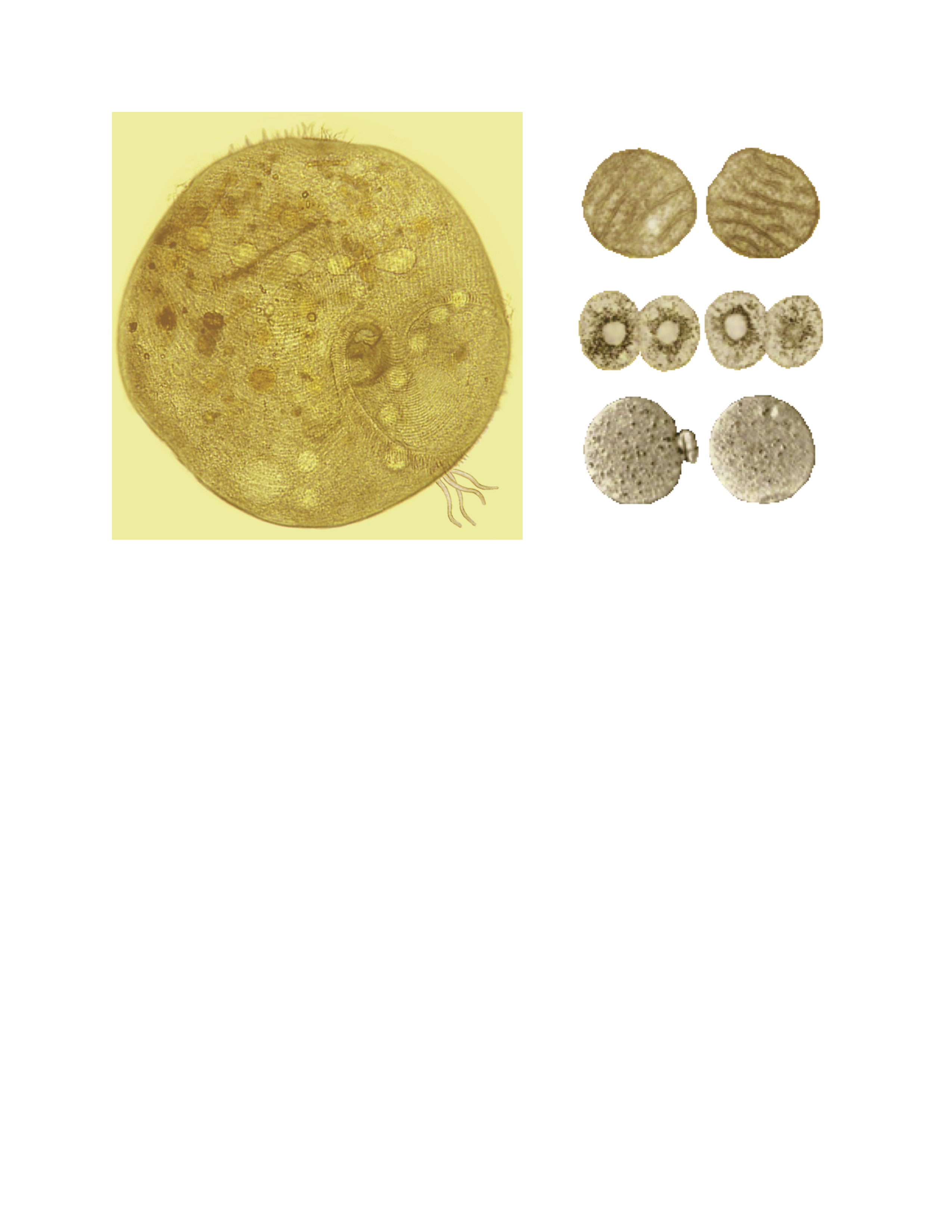

Supplement: Figure S2 — Stimulus and feature images for Experiments 2 and 7. The background (left) is located in the centre of a 1680×1050 resolution display, and the diameter of the approximate circle surrounding the background image approximately 1000 px. The full display was coloured yellow, like is shown behind the background cell. The one value of each of the three features (right) is pasted in the same locations as the features in Experiments 1, 9 and 10. The three features span approximately 80×80 pixels each. (TIFF) [file pone.0083302.s002.tiff]

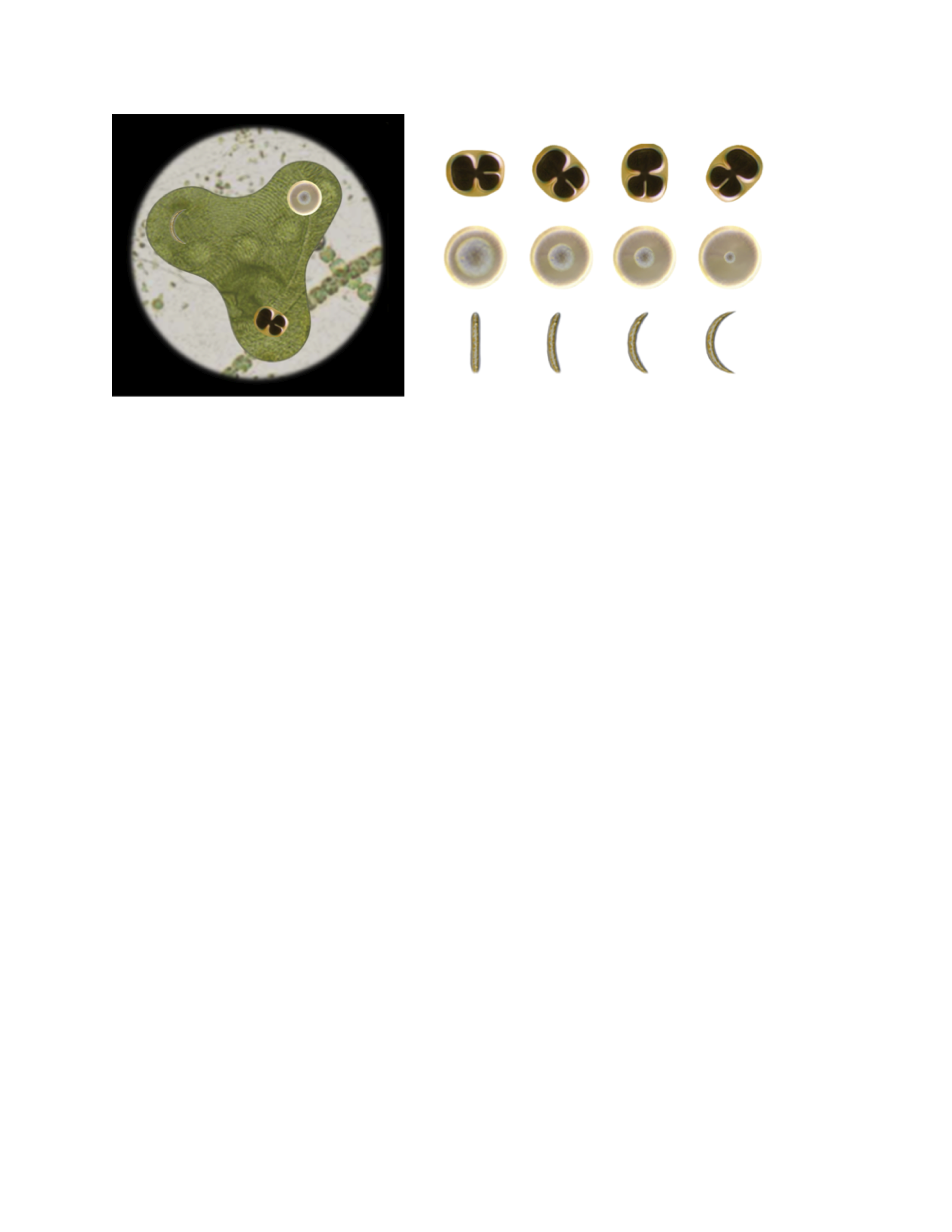

Supplement: Figure S3 — Stimulus and feature images for Experiments 3–6. The background (left) is located in the centre of a 1680×1050 resolution display, and the diameter of the circle surrounding the background image is approximately 1000 pixels. One value of each of the three features (right) is pasted in the arms of the fictitious microorganism. The three features span approximately 130×130 pixels each, and vary on one dimension over 90 degrees (see Figures 3–6A). The right side of the image shows examples of each of the three features at 0°, 45°, 60°, and 90° of variation of feature value. (TIFF) [file pone.0083302.s003.tiff]

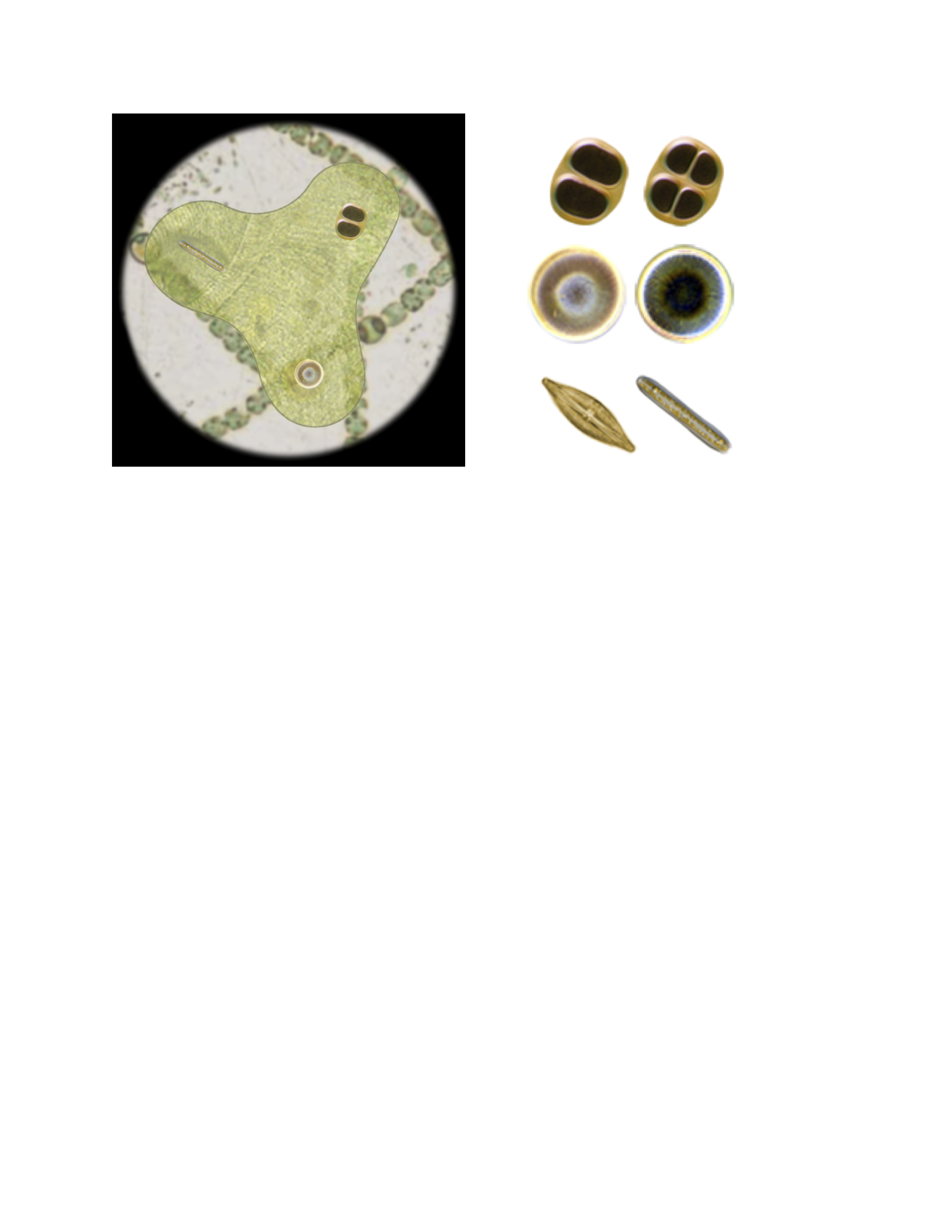

Supplement: Figure S4 — Stimulus and feature images for Experiment 8. The background (left) is located in the centre of a 800×600 resolution display, and the diameter of the circle surrounding the background image is approximately 590 pixels. One value of each of the three features is pasted in the arms of the fictitious microorganism. The three features span approximately 80×80 pixels each, and vary on one dimension taking on only two possible values. The left side shows one possible configuration of the features, all with one of the two possible feature values displayed. The location of each type of feature is constant for a single participant during the experiment, but the locations of the features are counterbalanced between subjects. (TIFF) [file pone.0083302.s004.tiff]
