# Supplementary material for: Learning-Induced Changes in Attentional Allocation during Categorization: A Sizable Catalog of Attention Change as Measured by Eye Movements
Source: PLoS One. 2014 Jan 31;9(1):e83302. doi: 10.1371/journal.pone.0083302 (PMC3908863; doi:10.1371/journal.pone.0083302)
Supplement: Table S1 — The category structures used in Experiments 3 and 4. For stimulus value tables, F1, F2, and F3 denote the stimulus dimension. The physical locations of the dimension on screen and the stimulus image used for the dimension are both counterbalanced. (DOC) [file pone.0083302.s005.doc]

|  | µ1 | µ2 | µ3 | µ4 | σ2 | covxy |
| --- | --- | --- | --- | --- | --- | --- |
| x | 45 | 22.5 | 67.5 | 45 | 65 | 0 |
| y | 22.5 | 45 | 45 | 67.5 | 65 | 0 |

Note. This was used to create stimuli for Experiment 4; the feature values in Experiment 3 were created by rotating these values by 45º. Here, μ = mean of each category; σ^2^ = variance; cov = covariance; x and y denote the x- and y-axis respectively. Each category was restricted within one of the quadrants defined by the equations y = x and y = -x + 90. Values for irrelevant features were generated by a normal distribution with a mean of 45 and standard deviation of 18.
