# Supplementary material for: Learning-Induced Changes in Attentional Allocation during Categorization: A Sizable Catalog of Attention Change as Measured by Eye Movements
Source: PLoS One. 2014 Jan 31;9(1):e83302. doi: 10.1371/journal.pone.0083302 (PMC3908863; doi:10.1371/journal.pone.0083302)
Supplement: Table S2 — The category structures used in Experiments 5 and 6. (DOC) [file pone.0083302.s006.doc]

|  | Category A | | | | |  | Category B | | | | |
| --- | --- | --- | --- | --- | --- | --- | --- | --- | --- | --- | --- |
|  | μx | μy | σx2 | σy2 | covxy |  | μx | μy | σx2 | σy2 | covxy |
| Exp 5 | 36 | 54 | 140 | 140 | 22.954 |  | 54 | 36 | 140 | 140 | 22.954 |
| Exp 6 | 30 | 45 | 12 | 120 | 0 |  | 60 | 45 | 120 | 12 | 0 |

Note. μ = mean; σ_x_^2^ = variation of distribution along the x-axis; σ_y_^2^ = variation of distribution along the y-axis; cov = covariance. Values for irrelevant features were generated by a normal distribution with a mean of 45 and standard deviation of 11.
