# Supplementary material for: Learning-Induced Changes in Attentional Allocation during Categorization: A Sizable Catalog of Attention Change as Measured by Eye Movements
Source: PLoS One. 2014 Jan 31;9(1):e83302. doi: 10.1371/journal.pone.0083302 (PMC3908863; doi:10.1371/journal.pone.0083302)
Supplement: Text S1 — Four Block Experiment Data. (DOC) [file pone.0083302.s007.doc]

**Text S1: Four Block Experiment Data**

The following data were used in the ANOVAs and post-hoc follow-ups.

|  | **Block 1** | **Block 2** | **Block 3** | **Block 4** |
| --- | --- | --- | --- | --- |
| **Accuracy** | .70 (.23) | .86 (.22) | .90 (.19) | .93 (.13) |
| **Number of Fixations per Trial** | 4.52 (2.82) | 3.23 (2.03) | 2.66 (1.41) | 2.02 (1.20) |
| **Probability of Fixating Irrelevant** | .35 (.29) | .19 (.32) | .12 (.27) | .07 (.16) |
| **Time Proportion Shift** | .71 (0.18) | .57 (0.19) | .41 (0.19) | .49 (0.28) |

Table 1. Block means (and standard deviations) for data from Experiment 1.

|  | **Block 1** | **Block 2** | **Block 3** | **Block 4** |
| --- | --- | --- | --- | --- |
| **Accuracy** | .61 (0.22) | .83 (0.20) | .92 (0.12) | .95 (0.06) |
| **Number of Fixations per Trial** | 6.22 (1.45) | 4.47 (1.30) | 3.88 (1.10) | 3.42 (0.96) |
| **Probability of Fixating Irrelevant** | .68 (0.27) | .37 (0.18) | .18 (0.15) | .08 (0.14) |
| **Time Proportion Shift** | .51 (0.12) | .43 (0.11) | .42 (0.13) | .40 (0.13) |

Table 2. Block means (and standard deviations) for data from Experiment 2.

|  | **Block 1** | **Block 2** | **Block 3** | **Block 4** |
| --- | --- | --- | --- | --- |
| **Accuracy** | .57 (0.16) | .75 (0.18) | .82 (0.15) | .84 (0.12) |
| **Number of Fixations per Trial** | 6.91 (2.55) | 4.84 (2.00) | 3.98 (1.76) | 3.35 (1.39) |
| **Probability of Fixating Irrelevant** | .67 (0.24) | .37 (0.35) | .22 (0.31) | .14 (0.25) |
| **Time Proportion Shift** | .49 (0.07) | .51 (0.14) | .49 (0.16) | .45 (0.16) |

Table 3. Block means (and standard deviations) for data from Experiment 3.

|  | **Block 1** | **Block 2** | **Block 3** | **Block 4** |
| --- | --- | --- | --- | --- |
| **Accuracy** | .59 (0.11) | .79 (0.11) | .82 (0.08) | .83 (0.09) |
| **Number of Fixations per Trial** | 6.39 (2.82) | 4.59 (1.85) | 3.66 (1.46) | 2.90 (1.08) |
| **Probability of Fixating Irrelevant** | .67 (0.28) | .35 (0.26) | .20 (0.30) | .12 (0.15) |
| **Time Proportion Shift** | .54 (0.11) | .56 (0.12) | .57 (0.17) | .58 (0.15) |

Table 4. Block means (and standard deviations) for data from Experiment 4.

|  | **Block 1** | **Block 2** | **Block 3** | **Block 4** |
| --- | --- | --- | --- | --- |
| **Accuracy** | .69 (0.12) | .80 (0.11) | .81 (0.08) | .82 (0.12) |
| **Number of Fixations per Trial** | 5.08 (2.21) | 3.66 (2.38) | 2.90 (1.84) | 2.75 (1.65) |
| **Probability of Fixating Irrelevant** | .63 (0.29) | .41 (0.35) | .26 (0.32) | .25 (0.33) |
| **Time Proportion Shift** | .43 (0.18) | .36 (0.19) | .34 (0.22) | .35 (0.23) |

Table 5. Block means (and standard deviations) for data from Experiment 5.

|  | **Block 1** | **Block 2** | **Block 3** | **Block 4** |
| --- | --- | --- | --- | --- |
| **Accuracy** | .88 (0.09) | .96 (0.04) | .97 (0.03) | .96 (0.04) |
| **Number of Fixations per Trial** | 4.37 (2.79) | 2.02 (1.51) | 1.66 (0.74) | 1.48 (0.67) |
| **Probability of Fixating Irrelevant** | .42 (0.29) | .12 (0.23) | .06 (0.16) | .04 (0.12) |
| **Time Proportion Shift** | .28 (0.20) | .17 (0.23) | .11 (0.23) | .06 (0.12) |

Table 6. Block means (and standard deviations) for data from Experiment 6.

|  | **Block 1** | **Block 2** | **Block 3** | **Block 4** |
| --- | --- | --- | --- | --- |
| **Accuracy** | .83 (0.53) | .92 (0.14) | .93 (0.12) | .96 (0.07) |
| **Number of Fixations per Trial** | 3.29 (1.45) | 1.86 (1.16) | 1.56(0.61) | 1.38 (0.46) |
| **Probability of Fixating Irrelevant** | .37 (.66) | .11 (0.25) | .06 (.17) | .03 (.07) |
| **Time Proportion Shift** | .32 (.18) | .10 (.17) | .07 (.19) | .04 (.12) |

Table 7. Block means (and standard deviations) for data from Experiment 7.

|  | **Block 1** | **Block 2** | **Block 3** | **Block 4** |
| --- | --- | --- | --- | --- |
| **Accuracy** | .47 (0.13) | .80 (0.15) | .96 (0.06) | .98 (0.04) |
| **Number of Fixations per Trial** | 8.82 (3.43) | 7.44 (2.74) | 5.96 (1.89) | 5.03 (2.06) |
| **Probability of Fixating Irrelevant** | .90 (0.11) | .82 (0.17) | .71 (0.22) | .61 (0.27) |
| **Time Proportion Shift** | .57 (0.15) | .57 (0.16) | .54 (0.12) | .54 (0.18) |

Table 8. Block means (and standard deviations) for data from Experiment 8.

| **Condition** |  | **Block 1** | **Block 2** | **Block 3** | **Block 4** |
| --- | --- | --- | --- | --- | --- |
| **1:1** | **Accuracy** | .67 (0.18) | .89 (0.12) | .95 (0.06) | .94 (0.03) |
|  | **Number of Fixations per Trial** | 4.99 (2.60) | 3.84 (2.15) | 3.09 (2.21) | 2.46 (1.49) |
|  | **Probability of Fixating Irrelevant** | .66 (0.17) | .50 (0.22) | .36 (0.22) | .35 (0.22) |
|  | **Time Proportion Shift** | .79 (0.15) | .76 (0.19) | .72 (0.21) | .70 (0.20) |
| **5:1** | **Accuracy** | .81 (0.09) | .95 (0.05) | .97 (0.02) | .98 (0.02) |
|  | **Number of Fixations per Trial** | 3.62 (1.12) | 2.65 (0.69) | 2.15 (0.11) | 1.69 (0.09) |
|  | **Probability of Fixating Irrelevant** | .42 (0.16) | .25 (0.12) | .21 (0.09) | .18 (0.08) |
|  | **Time Proportion Shift** | .74 (0.14) | .71 (0.14) | .65 (0.16) | .63 (0.16) |

Table 9. Block means (and standard deviations) for data from Experiment 9.

| **Condition** |  | **Block 1** | **Block 2** | **Block 3** | **Block 4** |
| --- | --- | --- | --- | --- | --- |
| **No Delay** | **Accuracy** | .76 (0.12) | .93 (.10) | .98 (0.05) | .99 (.01) |
|  | **Number of Fixations per Trial** | 4.31 (1.04) | 3.41 (0.96) | 3.13 (0.81) | 2.92 (0.62) |
|  | **Probability of Fixating Irrelevant** | .76 (0.22) | .43 (0.34) | .34 (0.32) | .27 (0.29) |
|  | **Time Proportion Shift** | .44 (0.13) | .36 (0.09) | .32 (0.08) | .29 (0.08) |
| **Delay** | **Accuracy** | .75 (.13) | .92 (0.11) | .98 (0.06) | .99 (0.03) |
|  | **Number of Fixations per Trial** | 2.68 (0.40) | 2.27 (0.38) | 2.24 (0.26) | 2.18 (0.24) |
|  | **Probability of Fixating Irrelevant** | .55 (0.23) | .20 (0.20) | .11 (0.17) | .08 (0.17) |
|  | **Time Proportion Shift** | .50 (0.16) | .36 (0.14) | .29 (0.12) | .26 (0.11) |

Table 10. Block means (and standard deviations) for data from Experiment 10.
